# Supplementary material for: Implementation processes in a cognitive rehabilitation intervention for people with dementia: a complexity-informed qualitative analysis
Source: BMJ Open. 2021 Oct 26;11(10):e051255. doi: 10.1136/bmjopen-2021-051255 (PMC8549661; doi:10.1136/bmjopen-2021-051255)
Supplement: Supplementary data [file bmjopen-2021-051255supp001.pdf]

Appendix 1: Methods and findings reported in accordance with the Standards for Reporting Qualitative Research (SRQR)

S1 Title	The title indicates the type of study and theoretical focus of the paper
S2 Abstract	An abstract with background, methodological detail and conclusions has been included
S3 Problem formulation	The introductory section comments on limitations of process evaluations and outlines how complexity theory could improve process evaluations
S4 Purpose or research question	The research question is stated in the first paragraph of the introduction.
Methods	
S5 Qualitative approach and research paradigm	The methods section states that the paper presents findings from a process evaluation embedded in a trial. The methods section describes the qualitative research as underpinned by a critical realist paradigm, and that qualitative interview and focus group data were analysed using a combined deductive and inductive approach.
S6 Researcher characteristics and reflexivity	The methods section states that the researchers analysing the data had no previous involvement in the study
S7 Context Setting/site and salient contextual factors	The introduction and background section provides information about the intervention setting, and includes references to more detailed publications about the study setting.
S8 Sampling strategy How and why research participants, documents, or events were	The methods section describes how participants were sampled. All available therapists were invited to a focus group, people with dementia were consecutively sampled across different sites, and therapy logs were sampled for around 10% of people receiving the intervention who had the best and worst outcomes from the intervention.

S9 Ethical issues	Details and reference number of the ethics approval are included
S10 Data collection methods	The timing and methods for data collection are described in detail in the methods section
S11 Data collection instruments	Data collection instruments are included in the appendices
S12 Units of study	Numbers and descriptions of participants are included
S13 Data processing	Transcription and use of NVivo are noted in the methods section. All data were stored on secure university servers.
S14 Data analysis	The method and stages of data analysis are described. The analysis of therapy logs drew on findings from the focus group – this is included in the description of methods.
S15 Techniques to enhance trustworthiness	The methods section states that focus group and interview data were collected by researchers without close involvement in the intervention. Data were analysed by researchers who had not been involved in the study up to that point (SMT and KW); developing themes from all datasets were discussed in meetings by the research team
Results/findings	
S16 Synthesis and interpretation	Findings are presented in relation to the theoretical concepts described in the introductory section.
S17 Links to empirical data	Quotes are included to substantiate the themes presented in the findings section
Discussion	
S18 Integration with prior work, implications, transferability, and contribution(s) to the field	Findings are summarised in the discussion section. Findings are also related to future implementation of the intervention and implications for the theoretical perspective (complexity theory)

Morgan-Trimmer S, *et al. BMJ Open* 2022; 11:e051255. doi: 10.1136/bmjopen-2021-051255

S19 Limitations Trustworthiness and limitations of findings	Limitations of the study are included in the discussion section.
S20 Conflicts of interest	The authors have declared there are no conflicts of interest.
S21 Funding	The funder and grant number is stated.
