# Supplementary material for: Implementation processes in a cognitive rehabilitation intervention for people with dementia: a complexity-informed qualitative analysis
Source: BMJ Open. 2021 Oct 26;11(10):e051255. doi: 10.1136/bmjopen-2021-051255 (PMC8549661; doi:10.1136/bmjopen-2021-051255)
Supplement: Supplementary data [file bmjopen-2021-051255supp002.pdf]

Appendix 2: GREAT focus group schedule - therapists' experience of providing goal-oriented cognitive rehabilitation for people with dementia (PwD) in GREAT

Introduction - aims of the focus group

The aim of this focus group is to explore therapists' experience of working with PwD to provide goal-oriented CR, to capture the nature of the interactions and the way in which the therapists deploy their skills to achieve the desired outcomes. We will use the results to inform the process of educating others on how to implement goal-oriented CR and to consider ways to further enhance CR in future.

- We value your open and honest responses. Please feel free to share your point of view even if it differs from what others have said. Every person's experiences and opinions are important.
- We are tape recording to capture everything you have to say, but we won't identify anyone by name in the report. The recording will be transcribed by someone from outside the team and anonymised.

General views on the process of providing goal-oriented cognitive rehabilitation – 10 min

How do you find the process of providing goal-oriented CR in GREAT?
What is it like to provide goal-oriented CR in GREAT?

Things that have worked well in providing goal-oriented CR – 10 min

What are the things that work well for you in the therapy process?
Which aspects do you look forward to most?

Challenges or difficulties in providing goal-oriented CR –10 min

What aspects of your work on providing goal-oriented CR in GREAT are most challenging?
What are the difficulties that you encounter when providing goal-oriented CR in GREAT?

Additional questions on the process of providing goal-oriented cognitive rehabilitation – 15 min

What sort of things have you learnt so far about working on cog rehab goals from working on the trial?
How do you find the role of the carer during the therapy process?
Has anything surprised you about the process?
Do you have hunches about what sort of people seem to benefit most?
If you were in charge, is there anything you'd be tempted to change about the way we have set up the study regarding working on the goals?

What do you think about the length of sessions?
What do you think about the overall length of the therapy?
How do you find training and supervision provided in GREAT?

If you were providing top tips to a therapist, what would be the key things you'd advise them to look out for or do?

Closing question

Before we finish the focus group, is there anything that we have missed? Is there anything anyone would like to say about their experience of providing goal-oriented CR in GREAT?

Morgan-Trimmer S, *et al. BMJ Open* 2022; 11:e051255. doi: 10.1136/bmjopen-2021-051255

Summing up and moving on

Summarise main points of the discussion, balance negative comments with positive comments. Thank everyone for sharing their comments on providing goal-oriented CR in GREAT and end the focus group.

Morgan-Trimmer S, *et al. BMJ Open* 2022; 11:e051255. doi: 10.1136/bmjopen-2021-051255
