# Supplementary material for: Implementation processes in a cognitive rehabilitation intervention for people with dementia: a complexity-informed qualitative analysis
Source: BMJ Open. 2021 Oct 26;11(10):e051255. doi: 10.1136/bmjopen-2021-051255 (PMC8549661; doi:10.1136/bmjopen-2021-051255)
Supplement: Supplementary data [file bmjopen-2021-051255supp003.pdf]

Appendix 3: Topics recorded by therapists in therapy logs

Therapy log topic	Therapist ratings or comments recorded under relevant sessions
Compliance	Whether given session was completed
Relationships	The relationship the therapist developed with the participant and carer, and the relationship between the participant and carer
Goals	Participant and carer responsiveness to the solution-focused problem-solving approach<br>Goal 1: agreed strategies, between-session practice, and progress<br>Goal 2: agreed strategies, between-session practice, and progress<br>Goal 3: agreed strategies, between-session practice, and progress<br>Any adjustments or modifications to goal statements provided at baseline<br>In-session goal attainment ratings by participant, carer and therapist (sessions 10 and 14) and comments on these<br>Selection of goal attainment scaling indicators for each goal (sessions 10 and 14) and comments on these
Activity levels	Review of activity levels, plan for behavioural activation to increase activity engagement, and comments on progress
Compensatory strategy use	Review of current use of compensatory strategies and environmental adaptations, plan to develop strategy use, and comments on progress
Restorative strategy use	Response to information about restorative strategies, plan for developing restorative strategy use, and comments on progress
Attention and concentration	Strategies introduced to help maintain attention and concentration, and progress with applying these strategies

Morgan-Trimmer S, *et al. BMJ Open* 2022; 11:e051255. doi: 10.1136/bmjopen-2021-051255

Anxiety management	Current use of anxiety management strategies, carer's perspective on the participant's use of these strategies, introduction or refinement of anxiety management technique(s), and progress with use of anxiety management strategies
Carer well-being	Review with carer, and plan for enhancing carer well-being
Carer involvement	The extent to which the carer was engaged in supporting the process of therapy
Ending therapy	Plans for maintaining progress after the end of the intervention, and review of other sources of help and support
Experience of therapy	Review with the participant and with the carer, and therapist reflection on the process of therapy; therapist confidence in addressing participants' goals (following the nine month follow-up)

Morgan-Trimmer S, *et al. BMJ Open* 2022; 11:e051255. doi: 10.1136/bmjopen-2021-051255
