# Supplementary material for: Implementation processes in a cognitive rehabilitation intervention for people with dementia: a complexity-informed qualitative analysis
Source: BMJ Open. 2021 Oct 26;11(10):e051255. doi: 10.1136/bmjopen-2021-051255 (PMC8549661; doi:10.1136/bmjopen-2021-051255)
Supplement: Supplementary data [file bmjopen-2021-051255supp004.pdf]

Appendix 4: Interview schedule for the person with dementia

The interview will take form of a conversation and the interviewer will encourage the participant to talk freely about the experience of the cognitive rehabilitation intervention.

The researcher will begin by re-establishing consent for the interview and for audio-recording.

The researcher may begin with some general conversation to build rapport as appropriate.

The researcher will introduce the main part of the interview by saying:
You've been taking part in the GREAT study and having visits from the therapist, and I'd like to know your views on what it was like. I'm interested in what it was like to take part in the study and how you found the visits from the therapist.

1. Experiences
The researcher will explore the participant's experiences and feelings starting with general questions:
How did you find the therapist's visits over the past few weeks?
What was it like to work with the therapist on your goals?

More specific information will then be elicited using prompts such as the following:
What were the more enjoyable things about your work with the therapist?
What were the less enjoyable things about your work with the therapist?
What aspects of your work with the therapist were more/less helpful?
Did you find it hard work to take part in the therapy?
What aspects of your work with the therapist were most challenging?
The interviewer will encourage the person to give specific examples, where possible.

2. Outcomes
The researcher will explore the impact of taking part in cognitive rehabilitation on the person's everyday life and self-perceptions.

What difference (if any) has your work with the therapist made to your daily life?
Has the experience changed anything in the way you think about your dementia/about yourself/about the future?
Has the experience changed anything in the way you relate to your carer/family?

The researcher will draw on positive comments from the participant to end the conversation on a positive note.
