# Supplementary material for: Implementation processes in a cognitive rehabilitation intervention for people with dementia: a complexity-informed qualitative analysis
Source: BMJ Open. 2021 Oct 26;11(10):e051255. doi: 10.1136/bmjopen-2021-051255 (PMC8549661; doi:10.1136/bmjopen-2021-051255)
Supplement: Author's manuscript [file bmjopen-2021-051255.draft_revisions.pdf]

BMJ Open is committed to open peer review. As part of this commitment we make the peer review history of every article we publish publicly available.

When an article is published we post the peer reviewers' comments and the authors' responses online. We also post the versions of the paper that were used during peer review. These are the versions that the peer review comments apply to.

The versions of the paper that follow are the versions that were submitted during the peer review process. They are not the versions of record or the final published versions. They should not be cited or distributed as the published version of this manuscript.

BMJ Open is an open access journal and the full, final, typeset and author-corrected version of record of the manuscript is available on our site with no access controls, subscription charges or pay-per-view fees (http://bmjopen.bmj.com).

If you have any questions on BMJ Open's open peer review process please email info.bmjopen@bmj.com

BMJ Open

Implementation as self-organisation: findings of the GREAT trial process evaluation from a complexity perspective

Journal:	*BMJ Open*
Manuscript ID	bmjopen-2021-051255
Article Type:	Original research
Date Submitted by the Author:	17-Mar-2021
Complete List of Authors:	Morgan-Trimmer, Sarah; University of Exeter, College of Medicine and Health<br>Kudlicka, Aleksandra; University of Exeter, College of Medicine and Health<br>Warmoth, Krystal ; University of Hertfordshire, School of Health and Social Work & NIHR Applied Research Collaboration East of England<br>Leroi, Iracema; Global Brain Health Institute, School of Medicine<br>Oyebode, Jan; University of Bradford, Centre for Applied Dementia Studies<br>Pool, Jackie; QCS Quality Compliance Systems, Dementia Pal Ltd<br>Woods, Robert; Bangor University, Dementia Services Development Centre Wales, School of Health Sciences<br>Clare, Linda; University of Exeter, College of Medicine and Health & NIHR Applied Research Collaboration South-West Peninsula
Keywords:	Dementia < NEUROLOGY, HEALTH SERVICES ADMINISTRATION & MANAGEMENT, QUALITATIVE RESEARCH

SCHOLARONE™
Manuscripts

I, the Submitting Author has the right to grant and does grant on behalf of all authors of the Work (as defined in the below author licence), an exclusive licence and/or a non-exclusive licence for contributions from authors who are: i) UK Crown employees; ii) where BMJ has agreed a CC-BY licence shall apply, and/or iii) in accordance with the terms applicable for US Federal Government officers or employees acting as part of their official duties; on a worldwide, perpetual, irrevocable, royalty-free basis to BMJ Publishing Group Ltd ("BMJ") its licensees and where the relevant Journal is co-owned by BMJ to the co-owners of the Journal, to publish the Work in this journal and any other BMJ products and to exploit all rights, as set out in our licence.

The Submitting Author accepts and understands that any supply made under these terms is made by BMJ to the Submitting Author unless you are acting as an employee on behalf of your employer or a postgraduate student of an affiliated institution which is paying any applicable article publishing charge ("APC") for Open Access articles. Where the Submitting Author wishes to make the Work available on an Open Access basis (and intends to pay the relevant APC), the terms of reuse of such Open Access shall be governed by a Creative Commons licence – details of these licences and which Creative Commons licence will apply to this Work are set out in our licence referred to above.

Other than as permitted in any relevant BMJ Author's Self Archiving Policies, I confirm this Work has not been accepted for publication elsewhere, is not being considered for publication elsewhere and does not duplicate material already published. I confirm all authors consent to publication of this Work and authorise the granting of this licence.

For peer review only

Implementation as self-organisation: findings of the GREAT trial process evaluation from a complexity perspective

Sarah Morgan-Trimmer, College of Medicine and Health, University of Exeter, St Lukes Campus, Heavitree Road, Exeter, EX1 2LU, UK. Email: s.morgan-trimmer@exeter.ac.uk (corresponding author)

Aleksandra Kudlicka, College of Medicine and Health, University of Exeter, Exeter, UK

Krystal Warmoth, Centre for Research in Public Health and Community Care (CRIPACC), School of Health and Social Work, University of Hertfordshire; NIHR Applied Research Collaboration East of England, UK

Iracema Leroi, Global Brain Health Institute, Trinity College Dublin, Dublin, Ireland

Jan Oyebode: Centre for Applied Dementia Studies, University of Bradford, UK

Jackie Pool, Dementia Pal Ltd, QCS Quality Compliance Systems, UK

Robert Woods, Dementia Services Development Centre Wales, School of Health Sciences, Bangor University, Bangor, UK

Linda Clare: College of Medicine and Health, University of Exeter, Exeter, UK; NIHR Applied Research Collaboration South-West Peninsula, UK

Wordcount: 5536

For peer review only

Abstract

Objectives: Health care is often delivered through complex interventions. Understanding how to implement these effectively is important for optimising services. This article demonstrates how the complexity theory concept of 'self-organisation' can inform implementation, drawing on a process evaluation within a randomised controlled trial of the GREAT intervention which compared cognitive rehabilitation for people with dementia with usual treatment.

Design: A process evaluation examined experiences of GREAT therapists and participants receiving the intervention, through thematic analysis of a focus group with therapists and interviews with participants and their carers. Therapy records of participants receiving the intervention were also analysed using adapted framework analysis. Analysis adopted a critical realist perspective and a deductive-inductive approach to identify patterns in how the intervention operated.

Setting: The GREAT intervention was delivered through home visits by therapists, in eight regions in the UK.

Participants: Six therapists took part in a focus group, interviews were conducted with 25 participants and 26 carers, and therapy logs for 50 participants were analysed.

Intervention: A 16-week cognitive rehabilitation programme for people with mild-to-moderate dementia.

Results: 'Self-organisation' of the intervention occurred through adaptations made by therapists. Adaptations included simplifying the intervention for people with greater cognitive impairment, and extending it to meet additional needs. Relational work by therapists produced an emergent outcome of 'social support'. Self-organised aspects of the intervention were less visible than formal components, but were important aspects of how it operated during the trial. This understanding can help to inform future implementation.

Conclusions: Researchers are increasingly adopting complexity theory to understand interventions. This study extends the application of complexity theory by demonstrating how 'self-organisation' was a useful concept for understanding aspects of the intervention that would have been missed by focusing on formal intervention components. Analysis of self-organisation could enhance future process evaluations and implementation studies.

Article Summary

Strengths and limitations of this study

- This study advances the application of complexity theory for evaluating health interventions, demonstrating how the concept of 'self-organisation' can be usefully applied in a process evaluation.
- Findings show how examining 'self-organisation' can help identify informal and less visible processes which are important for understanding how interventions operate.
- A limitation of the study is that it only reports therapist and participant perceptions of how the intervention operated; researcher observations of therapy sessions were not conducted.
- This study included a small number of focus group participants, interviewees and therapy records; the study was designed to produce theoretical generalisability through in-depth analysis rather than a large sample size.

For peer review only - http://bmjopen.bmj.com/site/about/guidelines.xhtml

For peer review only

Background

Introduction

This article discusses the application of the complexity theory concept of 'self-organisation' for understanding implementation. Implementation, defined here as *"the process through which interventions are delivered, and what is delivered in practice"* (1: 8), is an important element of process evaluation. This is distinct from the definition of implementation within the field of 'implementation science' which *"is the scientific study of methods to promote the systematic uptake of research findings and other evidence-based practices into routine practice"* (2: 1). It reports findings from a process evaluation which addressed several research questions, including "how was the intervention implemented?". Process evaluations may be the first opportunity to closely study, refine and optimise implementation of a new intervention. Findings on implementation can then inform wider roll-out of interventions at the post-trial stage (1).

Existing approaches to implementation employed by process evaluations face two main limitations. First, process evaluations are often structured using logic models which represent the underlying theory of how an intervention, including implementation processes, work (3). Logic models usefully provide a simple-enough model on which to base an evaluation, but in doing so they tend to conceptualise the world in a relatively linear, mechanistic way. Second, logic models focus attention on whether a certain process happened, rather than unexpected events, because they represent ideal versions of what researchers hypothesise will occur. Within this approach, implementation of the intervention tends to be examined in terms of fidelity to this ideal version. However, during implementation, some components of the intervention may not have been delivered as anticipated, additional components may have been introduced, and components may have interacted with each other or contextual factors in unexpected ways. These aspects may be missed if data are only collected on components identified in logic models. This article discusses the application of the complexity theory concept of 'self-organisation' for improving understanding of how complex health interventions are implemented. It examines how it can be applied through analysing process evaluation data from a trial of a community-based cognitive rehabilitation (CR) intervention for people with dementia (4, 5).

Complexity theory and self-organisation

Complexity theory has been increasingly advocated as an alternative theoretical lens for understanding complex interventions, and is being increasingly applied in evaluation studies (6-10). It theorises systems of different types – social, physical, biological – as constantly self-organising and unfolding. A complex system cannot be understood only in terms of its parts, as a complicated machine for example, where the causal powers of the system are explained by its components (6, 9, 11). Processes occur in complex systems through the dynamic, non-linear relations between components at different levels as interacting parts of a system, producing effects in unpredictable ways (12, 13). Complexity theory encompasses a range of concepts which describe how patterns occur, including self-organisation, feedback loops and emergence (10, 12-14). Complex health interventions can be conceptualised as complex systems since they incorporate multiple interacting

For peer review only - http://bmjopen.bmj.com/site/about/guidelines.xhtml

elements such as humans (as active and responsive agents), organisations, materials, rules, policies and so on, through which these patterns may occur (6, 11, 12). Complexity approaches also conceptualise the system within which an intervention is introduced as complex, unfolding, interacting with and changed by the intervention: systems are nested within other systems and cannot be isolated (6, 13-16).

This article draws on the complexity concept of 'self-organisation', and the additional concepts of 'emergence' and 'feedback loops', and explores their application to studying implementation. 'Self-organisation' refers to interacting elements of a system, such as practitioners, who behave or make individual micro-level decisions in response to other elements of a system (such as clients) in a way which produces patterns or order at a higher level (10, 13). This order is produced in a bottom-up way since it occurs as the result of individual decisions which are not coordinated, rather than through top-down instructions. The order which is created is 'emergent', as it appears at the overall intervention level and occurs somewhat unpredictably. A common form of self-organisation occurs through feedback loops, for example where practitioners tailor how they deliver an intervention in response to client reactions to the intervention (11).

Self-organisation can operate within or beyond the formal design of an intervention. Some interventions are designed to be partly self-organising, where the practitioner is expected to adapt the intervention according to individual client needs for example, as long as the core functions of the intervention are delivered (17, 18). Other forms of self-organisation occur outside of the prescribed parameters of the intervention, through deviation from protocols for example. Through micro-level decisions about adaptations, practitioner behaviours result in self-organisation as their patterns of behaviour at a collective level shape what intervention is actually delivered. Self-organisation is not commonly theorised as being part of implementation processes, yet could significantly influence implementation and, in turn, intervention outcomes.

While complexity theory may explain the real world more adequately than linear models, there are challenges in applying it within a process evaluation. Complexity theory is a collection of related concepts which are often presented in overlapping rather than standardised lists, and does not have an agreed definition (10). It is not yet a consolidated theory in that there are questions about how concepts are related to each other (9, 14). Complexity theory also tends to emphasise the unpredictability of outcomes and produce post-hoc explanations rather than having strong predictive power (10, 19). This is somewhat at odds with the aim of process evaluation, and research more generally, to identify generalisable patterns, 'demi-regularities' or predictive theories (6). However, the emphasis on unpredictability varies within the family of complexity theory approaches (6). This article examines the application of complexity concepts, particularly 'self-organisation', in a process evaluation and reflects on its use for understanding the implementation of complex health interventions.

The GREAT trial

The GREAT study was a randomised controlled trial, with an embedded process evaluation, of a CR intervention for people with a clinical diagnosis of dementia (referred to here as 'participants') and a Mini Mental State Examination (MMSE) score of ≥ 18 points indicating mild to moderate cognitive

For peer review only - http://bmjopen.bmj.com/site/about/guidelines.xhtml

impairment (20, 21). It was delivered in eight regions in the UK. The main purpose of the intervention was to improve ability to carry out everyday activities in the areas the participant chose to target. The intervention involved identifying with the participant personally relevant and significant goals related to daily activities, and then working together to develop and implement a set of strategies to enable the person to achieve the desired outcomes. The primary outcome was participant-reported progress towards participant-identified goals at three months. It was delivered by a practitioner (eight of whom were occupational therapists and one a nurse) during one-hour home visits, ideally with a carer also present at part of the session. The participant, with carer support, was encouraged to implement agreed changes in daily routines and practise strategies between visits. Ten visits were delivered over three months, followed by four maintenance sessions over six months. The intervention was detailed in a practitioner handbook which included a structured protocol (22); the core component of sessions was to work towards personalised goals, supplemented by strategies to improve attention and concentration, compensatory strategies, and restorative strategies for retaining new information or improving recall. Additional optional components included anxiety management, increasing activity levels, discussing carer well-being, and signposting to other services. Training sessions and regular group and individual supervision was provided for the therapists. Full details of the intervention, trial methods and trial outcomes have been published (4, 5, 20, 23).

The intervention was personalised in that participants could identify up to three goals they wished to work towards and the therapists applied CR strategies to address these. The intervention was also designed to be responsive to participant needs in that therapists had some flexibility to apply elements of the intervention such as anxiety management, depending on the needs of the participant and their personal and social contexts. The handbook reflected this balance between structure and flexibility, for example:

> *The intervention in a trial needs to follow a structured protocol, as summarised in Table 1 below. However, some flexibility will be needed as participants will have varying needs and preferences and will progress at different rates.*
> GREAT Handbook for Therapists (22: 40)

This article examines the implementation of the GREAT intervention, where the intervention is conceptualised as a complex intervention designed to incorporate a degree of self-organisation through its person-centred design and requirement to be responsive to the contexts, needs and preferences of people with dementia.

Methods

This article draws on findings from three datasets which were analysed for the GREAT trial process evaluation (7). Methods and findings are reported in accordance with the Standards for Reporting Qualitative Research (SRQR) checklist (Appendix 1). A focus group and intervention therapy logs examined the perceptions and experiences of therapists, and a set of interviews explored the perceptions and experiences of participants and their carers. Written informed consent was obtained at the beginning of the trial. Trial researchers and therapists were trained to monitor ongoing consent and identify and respond to any indication of possible

For peer review only - http://bmjopen.bmj.com/site/about/guidelines.xhtml

withdrawal of consent. Researchers began participant and carer interviews by re-establishing consent.

Focus Group

A focus group was conducted with six of the nine therapists at the end of the first year of the intervention to examine their experiences of the intervention (see Appendix 2 for schedule). The focus group was conducted as part of the second annual training event for the trial therapists. The discussion was facilitated by co-author and trial co-investigator JO. She had a thorough understanding of the intervention but was not involved in supervising the therapists, which facilitated an open discussion. It was digitally recorded, transcribed verbatim and uploaded to NVivo 11. A thematic analysis, underpinned by a critical realist perspective, was conducted by the lead author, SMT, who had not been involved in the study up to this point (24). Data were initially coded using a combined inductive-deductive approach, drawing on the dementia literature informing the GREAT trial (4). Themes were then developed, after first summarising the codes, and graphics were developed to represent and analyse themes at a more interpretive level. This article draws on three themes relevant to implementation: the perceived influence of the severity of dementia; adaptation work of therapists in response to the severity of dementia; and the relational work conducted by therapists.

Analysis of therapy logs

Therapy logs were maintained for every participant for each of the 14 sessions and included detail about each intervention component, such as progress towards goals (see Appendix 3 for categories included in therapy logs). These logs were analysed by SMT to try to identify factors which might explain how participants benefitted from the intervention, or not. Therapy logs were analysed to compare the 25 participants with the best primary outcomes (the 'good outcomes' group) and the 25 participants with the poorest primary outcomes (the 'poor outcomes' group), out of the intervention arm population of 281 individuals.

An adapted framework analysis method was employed, using deductive categories identified in the focus group findings and also inductive categories where novel factors were identified (25). The analysis aimed to identify differences in patterns in each group, paying attention to both qualitative and quantitative differences. First, each component for each session was summarised and compared between the 'best outcomes' and 'poor outcomes' groups. Second, the trajectory of each participant's progress through the intervention was summarised and then compared by group. Third, a 'negative case analysis' was conducted to explore factors which did not fit with the general patterns emerging from the first two stages of the analysis.

Interviews

Face-to-face, semi-structured interviews were conducted with 25 participants and 26 carers at nine months post-intervention, to explore their experiences of the intervention (see Appendix 4 for interview schedule). They were consecutively sampled across three different sites sites (North Wales, South Wales and Greater Manchester) where the intervention was taking place and where there was a research assistant not otherwise involved in the trial available to complete the interviews. Participants and carers were interviewed separately wherever possible, starting with the person with dementia. Interviewers took a photograph of the therapist on the visit to prompt the

For peer review only - http://bmjopen.bmj.com/site/about/guidelines.xhtml

participant's memory of the therapy sessions. If the participant was struggling to recall the therapy sessions, the interview was completed jointly with the carer. All interviews were audio-recorded, transcribed verbatim and uploaded to NVivo 11 (4).

Data were analysed using thematic analysis which was underpinned by a critical realist position and which employed an inductive approach to identifying and exploring patterns of meaning (26, 27). Data were initially coded by four researchers, and then organised into meaningful groups by KW and SMT. Related themes were clustered together and organised into an overall thematic map.

Detailed methods and findings of the focus group, therapy log analysis and interviews have been reported (4). In a last stage of analysis, themes from the focus group analysis were interpreted in relation to core concepts in the complexity literature(4, 10). These themes were then further developed through triangulation with findings from the interviews and therapy log analysis. Findings on intervention implementation are reported below.

Results

Findings presented here explain how the intervention operated as a complex system, and how some aspects of implementation occurred in a self-organising pattern, through adaptation behaviours of therapists. These were: simplifying the intervention for people with greater cognitive impairment; providing additional support in response to participant needs; and conducting relational work to engage participants, which produced an emergent outcome of increased social support.

Self-organisation through adaptive response

The GREAT intervention incorporated self-organisation in its design: therapists were required to select CR strategies appropriate for individuals and their social context, and tailor the intervention to individuals' needs and preferences. Participants and carers reported experiencing the intervention as personalised:

> *[Goals] were always relevant to … obviously relevant to the issues that [therapist] wanted to raise … And also relevant to, the issues that were important for [person with dementia] … she worked at a pace that was good for him as well.*
> (Interview, Carer 5)

Therapists reported that they adapted the intervention in response to their perception that participants with greater cognitive impairment (within the trial population range) were less likely to engage in and benefit from the intervention. For example, therapists observed that these participants had more difficulty setting relevant goals or remembering them. Therapists' notes for participants in the 'poor outcomes' group also described features which could be attributable to greater cognitive impairment: a tendency to set more basic-level goals; being more likely to give up on a goal; lower levels of motivation; being more withdrawn during sessions; and having less awareness regarding their condition.

For peer review only - http://bmjopen.bmj.com/site/about/guidelines.xhtml

Therapists responded to participants with greater cognitive impairment through adapting their delivery of the intervention for these participants in several ways. First, because it was difficult for these participants to absorb information, therapists would slow down the pace of delivery and only deliver what they thought were the most relevant sections:

> *Therapist 5: I was almost going at a snail's pace, because I realised it was far too much information to dish out, and I was literally cherry-picking the bits I thought were relevant so I could get through the goals and get them rated, and just literally [Several people agreeing]*
> *Therapist 4: And, sometimes you do have to cherry pick don't you? [Several people agreeing].*
> (Therapist focus group)

Therapists also tailored the timing of material, moving some supplementary topics to an alternative session for example:

> *Therapist 6: But there's too much information for me, so let alone someone who has to take a while, or so I've started moving things away*
> *Therapist 4: Oh, I've moved things very early on, I moved the anxiety stuff, unless it's the glaring, big problem, I move anxiety from week 1 to week 2 because it's too much information.*
> (Therapist focus group)

In addition, therapists reported that they simplified some of the language in the handbook:

> *Therapist 7: The 'restorative' and 'compensatory', that's too jargon-y, and too heavy for the person, not all, but some of the people with dementia…*
> *Therapist 2: Yes, I've had to change the words to 'methods', because 'strategies' just scares them.*
> (Therapist focus group)

In these ways, therapists made individual, micro-level decisions to ensure the intervention matched the needs of participants with greater cognitive impairment, and sometimes adapted the content from the intervention protocol (22). This is an example of self-organisation operating in a feedback loop, where practitioners adapted an intervention according to the perceived capacity of people with dementia. Through this adaptation work, they shaped the nature of intervention for some participants.

Self-organisation through extending the intervention

Carers were important for the delivery of the intervention because they supported the practice of new techniques, and could also impact on participant motivation. Difficulty engaging some carers was noted in the therapy logs and, in the focus group, therapists reported additional efforts they made to engage carers. For example, therapists sometimes adjusted the time allocated (often increasing it) for home visits and also changed the timing of visits to ensure they met with carers:

> *Therapist 7: I mean I've done home visits at 8 o'clock in the evening, just to catch up with the carer, so I can have face-to-face and actually get what's going on, because I can't get hold of*

For peer review only - http://bmjopen.bmj.com/site/about/guidelines.xhtml

her during the day, she just doesn't answer her phone. So, having to do home visits in the evening, so that's impacting, that's where the hours are coming in, from doing more of that work…
Therapist 6: I've often gone out on home visits, and they've [the carer has] gone out. Then you have to extend your visit by an extra 20, 30 minutes because, you don't want to just leave… So you have to extend your visit sometimes, or you have to ring [the carer] afterwards.
(Therapist focus group)

Therapists sometimes also had to take additional time to identify and meet additional carers who were most likely to be the person supporting the intervention but not the carer who was originally nominated to take part in the intervention:

Therapist 4: Actually the workload increases for us, because what we're doing is we're talking to the carer that's on the spot, and we're also having to liaise with the son and daughter, either by text or email, or something else in the evenings, that has quite a massive implication [agreement from others].
(Therapist focus group)

Therapists also delivered several 'add-on' components in response to the needs of participants. First, the intervention component to address carers' levels of well-being was formally limited in the handbook to referring carers to local sources of support: *You can direct carers to appropriate sources of support in the local area, and encourage them to access these (22: pg. 51)*. However, one therapist reported contacting services directly:

Therapist 7: I'm also contacting social services, so that the carer will get a break, that shouldn't be part of my role, but no-one else seems to.
(Therapist focus group)

Second, therapists commented on (often marital) conflict in the relationships between participants and their carers, also noted in therapy logs, and attempted to reduce conflict even though they were aware it was beyond the parameters of the intervention:

Therapist 2: I do find that it's about the nature of the relationship as well. And often you do find, like you're doing a couples intervention, it's not just about the dementia, it's often about the dynamics that have probably gone on through their whole relationship but the situation is highlighting it, and that's really quite difficult to manage, isn't it?
Therapist 6: Sometimes it's like marriage counselling
Therapist 2: Yeah… it's about their relationship, isn't it, it is a bit of the couples stuff, and I find myself doing that, and I think it is helpful, but it's beyond what we've been given.
(Therapist focus group)

Several carers described in interviews how the therapist acted as a mediator in conflicts between the carer and person with dementia. Carers also reported improved relationships between themselves and participants as a result of the intervention, for example having increased understanding of and

For peer review only - http://bmjopen.bmj.com/site/about/guidelines.xhtml

patience with the person with dementia. Several participants commented that they had greater social awareness and were more likely to consider the impact of what they said on others.

In these ways, self-organisation occurred through therapists' micro-level decisions to adjust (often increase) the time taken to engage carers, directly contact services, and reduce conflict between carers and people with dementia. Some therapists extended the intervention so that it was larger in scope than described in the handbook. These patterns of self-organisation can also be understood in term of feedback loops, where therapists' adaptive behaviours were in response to the contexts and unmet needs of people with dementia and their carers.

Self-organisation in relational work and emergent 'social support' outcomes

Therapists engaged in building relationships with participants and their carers; the intervention handbook described one of the therapist's roles as '*Developing rapport with participants and carers and building good relationships' (22: pg. 42)*. In interviews, people with dementia described positive relationships with therapists:

> *Oh fine, yeah fine, got on well … Easy, yeah she explained everything and, you know, it was no hardship (laughs)…That's right, yeah, well sometimes when people come to see you, … you're afraid to talk, you know, afraid to say anything when it's a little bit dumb. But she made me feel so comfortable and within a couple of minutes we were just like as though we'd been friends for a long time.*
> (Interview, Person with dementia 1)

Therapists reported that relationships with participants were an important aspect of the intervention, helping to engage people with dementia in the intervention and motivate them:

> *Therapist 2: The actual nature of the relationship and that therapeutic rapport, which I think actually counts for a lot, but I think it largely goes unmeasured in a way, what we bring as people, and our relationship, and that's the motivating factor, and it's hard to know how you would measure that, but I think there is a lot about them getting to know you and you becoming part of their routine, and that goes a long way*
> *Therapist 5: I think they open up don't they*
> (Therapist focus group)

A good relationship was also important for therapists' work in that getting to know participants well underpinned their ability to help participants identify relevant goals and develop personalised strategies.

Additionally, therapists recognised that their relational work and the provision of social support as being important in itself and would dedicate time specifically to this aspect of their visits:

> *Therapist 6: I think sometimes it's that really good for the carer and the participant to have somebody going in and for such a long time. You do work on the goals, but another big part of it is quite, you know, supportive, and you know, social, because you spend the first ten minutes*

For peer review only - http://bmjopen.bmj.com/site/about/guidelines.xhtml

For peer review only

of your visit, they just go on about what they've done in the past week, and you can't be like, come on let's crack on [laughter] I think you do see in a lot of people, especially as you go on, they thaw a bit as you visit and then [several people agreeing] I get that good bond by session ten, and you're sad to pull out, but I think that support does really help.
(Therapist focus group)

This relational work was self-organising in that therapists conducted relational work and provided social support, going slightly beyond the intervention parameters by regarding it as an intervention component in itself and spending more time on this than reflected in the handbook.

The social support provided by the therapists also developed into an emergent outcome of the intervention in that it was perceived as the main benefit for some participants:

Therapist 7: the pleasure of seeing people try and do well, even if they're not achieving their goals, it's the other things that they're getting from it, the social interaction, the time to talk about their condition, the dementia, and it not being hushed away and in the cupboard.
(Therapist focus group)

In interviews, some people with dementia were unable to recall the goals they had been working towards but many commented on the relationship with the therapist and that they would miss the visits now the therapy had ended. Carers also commented on relational benefits for people with dementia:

I think my mum just enjoyed it more that somebody was, the social aspects of it, that somebody was coming
(Interview, Carer 1)

The therapy log analysis indicated that relational and social support outcomes were an important (or at least the best-recalled) element of the intervention by people with greater cognitive impairment. At the end of the intervention, participants from the 'poor outcomes' group were more likely to refer to the relational or social aspect of the therapist visiting them as a positive element of the intervention, whereas participants in the 'best outcomes' group were more likely to give examples of formal components of the intervention that had benefitted them. One therapist also commented in the focus group that social support could be the primary benefit for the carer.

In these ways, relational work conducted by therapists and their provision of social support extended the scope of intervention as it was described in the handbook. Self-organisation occurred through micro-level decisions of therapists which helped them deliver the intervention effectively but also meet the social support needs of participants and in doing so developed into an emergent outcome.

Discussion

This article provides an example of how process evaluation findings can be interpreted using complexity theory and the concept of self-organisation. Some implementation processes occurred as

For peer review only - http://bmjopen.bmj.com/site/about/guidelines.xhtml

self-organisation, through individual decisions made by therapists. These decisions produced 'order' at a higher level by creating patterns of service delivery and outcomes. The self-organisation of the intervention occurred partly by design, through a person-centred approach, but also through adaptions (to severity of dementia), extensions (of therapists' time and social support) and add-on components (carer support and relationship conflict resolution) (28). The handbook recommended but did not include detail about the adaptation of the intervention, and also implied flexibility in relation to the delivery of formal components rather than extending the time allocation or scope of the intervention (22). It was not always clear, therefore, where self-organisation fell within or outside of the intervention parameters. Some of the adaptations resulted from therapists' interpretation of the flexibility allowed within the intervention as an aspect of its personalised approach, to achieve participants' goals which was the core component of the intervention. Therapists are likely to have drawn on their professional training and experience to make decisions about when and how to adapt the intervention. For example, skilled therapists such as OTs would generally translate complex material for use with clients, such as simplifying language in a handbook. Therapists also felt they were going beyond the intervention design at times, particularly in feedback loops when they provided 'add-ons' in response to participants' needs and contexts such as addressing relationship conflicts. Implementation within a trial context meant the intervention was described in a structured way in the handbook and there was less flexibility than would be the case in normal clinical practice. This may have led therapists to view the intervention as relatively structured and been more likely to view their adaptations as falling outside the intervention parameters.

The findings about self-organisation have several implications for further studies or wider roll-out of the GREAT intervention. First, the adaptations made by therapists for people with greater cognitive impairment (within the trial range of a Mini Mental State Examination score of ≥ 18 points) could be added to the handbook as examples of tailoring to the specific needs of the person, and inform training for therapists. Second, referrals to other services and specialised professionals could be enhanced in order to meet the wider needs of people with dementia and their carers, particularly for common issues such as relationship conflict. Third, social support needs could be addressed by referral to an alternative, less resource-intensive intervention such as befriending. Alternatively, the emergent outcome of social support, particularly for those with greater cognitive impairment, could be more formally incorporated into the intervention design. This may require increased resourcing, including formally incorporating this skill into recruitment and training for professionals, depending on what prior training practitioners have received (29). This intervention was delivered mainly by skilled OT therapists who typically offer a range of types of support; if the intervention was delivered by OT assistants, for example, additional training might be required. This also depends on whether future interventions are defined as targeted CR interventions or expanded to incorporate the additional elements observed in this study. Fourth, the time allocation for visits could be revised, or made more flexible, to accommodate the additional time requirements for some visits due to the extended or 'add-on' components provided. Lastly, future evaluations of the intervention could investigate the impact of social support for participants and carers, since findings indicated this area was important but an in-depth examination of this was beyond the scope of the study.

For peer review only - http://bmjopen.bmj.com/site/about/guidelines.xhtml

This analysis had several limitations. First, the qualitative data are the views and perceptions of the therapists, participants and carers of how the intervention operated, and the therapy logs were relatively brief notes made by therapists. Second, only one focus group of six therapists was conducted, and the therapy log analysis was limited to 50 participants. However, this analysis was designed to produce theoretical generalisability through in-depth analysis rather than a large sample size.

Applying a complexity theory lens had advantages over traditional process evaluation approaches. It explained how decisions dispersed throughout a system at the micro-level interacted with population characteristics and context, and how this influenced what intervention was delivered and what outcomes it produced. This type of self-organising feedback loop is likely to be a common pattern in health interventions, where practitioners adapt interventions to meet the needs of their clients. It may be particularly common where participants have high levels of unmet needs, co-morbidities or in complex cases. Future studies of implementation could analyse the micro-processes of self-organisation specifically, in addition to formal components of interventions. This type of approach requires an agnostic position on fidelity of intervention delivery since lack of adherence to intervention protocols is not necessarily a negative aspect of the intervention. Although some degree of fidelity is important for studies such as trials, understanding how adaptation may occur is important for real-world implementation. Adaptive behaviours could be positive in that they support individuals, help engage participants, and help tailor the intervention to a local context (12). However, adaptive behaviours could also create difficulties if they expand the scope of the intervention beyond what is possible for sustained delivery, or are ineffective. Expansion could displace the delivery of core components of the intervention, for example, or lead to burnout in practitioners. Clarity about the balance required between fidelity and adaptation is therefore necessary (30).

A complexity perspective was also useful in that it identified informal, less visible processes which might not be picked up by standard evaluation models measuring fidelity against formal components and protocols. One particularly informal and non-visible aspect of the intervention was the relational work conducted by therapists and the provision of social support through this. Findings from this study were that it was an important aspect of how the intervention operated: it facilitated participant and carer engagement in the intervention, supported aspects such as personalisation, and underpinned improved social support valued by participants and their carers. While relational and social support work may be recognised by practitioners as a common micro-level process, it is given comparatively little attention in published health intervention research (31, 32). This study suggests it is worthy of more attention in health intervention evaluation studies.

Complexity theory was used here to retrospectively explain the implementation of an intervention, which is less useful than predictive theory. However, the latter is challenging (10, 33). Rather than being predictive in a strong sense, complexity theory could inform programme theories and logic models to include commonly-occurring patterns such as self-organisation or feedback loops (34). This could be supported through developing less linear logic models and applying them flexibly as programme theory develops during an evaluation (3, 16). To this end, complexity theory could be more useful for process evaluation as a methodological theory to guide modelling, data collection and analysis, or an overarching framework, rather than a type of theory that explains how the world

For peer review only - http://bmjopen.bmj.com/site/about/guidelines.xhtml

works in a strongly predictive sense (9, 35). An alternative approach is to combine complexity approaches with other theoretical perspectives which explain agency or structures at different levels of a system (6, 8, 36). Both approaches require multiple, exploratory and flexible methods, including qualitative methods of sufficient depth, in order to identify informal, complex and unpredictable patterns (6, 7, 37). While complexity theory is increasingly employed, calls have been made for further examples of its application in process evaluation (1, 38). This article has provided one example of how complexity theory, particularly the concept of self-organisation, can be useful for providing insight into the implementation of an intervention that would have been missed by a process evaluation only focusing on formal intervention components.

Author contributions

SMT designed and conducted the analysis of the focus group and therapy log data, oversaw the analysis and discussed themes from the interview data; and devised and drafted the paper; OK discussed themes developed from the focus group and therapy log data, and also commented on several drafts of the paper; KW analysed interview data and commented on several drafts of the paper; JO conducted the focus group, contributed to the study design and commented on several drafts of the paper; II, JP, and BW contributed to the study design and commented on several drafts of the paper; LC oversaw the study design, discussed themes developed, and commented on several drafts of the paper.

Disclaimer

KW is now supported by the NIHR Applied Research Collaboration East of England. The views and opinions expressed therein are those of the authors and do not necessarily reflect those of the HTA programme, Applied Research Collaboration South West Peninsula, Applied Research Collaboration East of England, NIHR, NHS or the Department of Health and Social Care.

Acknowledgements

We thank all the participants with dementia and their family supporters who generously contributed their time to be part of this study. We also thank our experts by experience and staff at Alzheimer's Society for supporting the study. We would like to acknowledge the key roles played by Ho Yin Chan, Matthew Lewis and Julie Nixon who conducted the qualitative interviews, Dr Gill Toms who conducted some of the qualitative data analysis, and the trial therapists who provided the intervention.

Patient and Public Involvement

For the GREAT trial, in which the process evaluation was embedded, experts by experience including Alzheimer's Society Research Volunteers were consulted at the set-up stage to inform participant information resources and trial procedures. As a result we made a number of amendments to the participant-facing documents and assessment measures. The inclusion of qualitative interviews and a focus group in the study design was at the suggestion of the experts by experience. They also provided insightful comments about the progress of the trial and contributed to developing a follow-up application for an implementation grant. Study participants were updated about trial progress through a regular newsletter.

For peer review only - http://bmjopen.bmj.com/site/about/guidelines.xhtml

Trial registration

ISRCTN21027481

Ethics approval statement

The study was reviewed by Wales Rec 5, which issued a favourable opinion on 25th June 2012 (Reference 12/WA/0185), and was approved by the Bangor University School of Psychology Research Ethics Committee.

Reporting statement

Methods and findings are reported in accordance with the Standards for Reporting Qualitative Research (SRQR) checklist (see appendix 1).

Competing interests statement

The authors declare they have no competing interests.

Funding statement

This work was supported by NIHR HTA, grant number 11/15/04.

Data sharing statement

Interview and focus group data were not deposited in an archive due to the small numbers of individuals participating in named sites, which could compromise anonymity through potentially identifiable information in transcripts. Therapy logs data were clinical notes and therefore not appropriate for depositing in an archive.

References

1. Moore G, Audrey S, Barker M, et al. Process Evaluation of Complex Interventions: Medical Research Council Guidance. London: MRC Population Health Science Research Network; 2014.
2. Eccles MP, Mittman BS. Welcome to Implementation Science. Implementation Science. 2006;1(1):1.
3. Rogers PJ. Using programme theory to evaluate complicated and complex aspects of interventions. Evaluation. 2008;14(1):29-48.
4. Clare L, Kudlicka A, Oyebode JR, et al. Goal-oriented cognitive rehabilitation for early-stage Alzheimer’s and related dementias: the GREAT RCT. Health Technol Assess. 2019;23(10).
5. Clare L, Kudlicka A, Bayer A, et al. Goal-oriented cognitive rehabilitation in early-stage Alzheimer's and related dementias: results from a multi-centre, single-blind, randomised controlled trial (The GREAT trial). Alzheimer's & Dementia: The Journal of the Alzheimer's Association. 2017;13(7):P899-P900.
6. Walton M. Applying complexity theory: a review to inform evaluation design. Eval Program Plann. 2014;45:119-26.
7. Greenhalgh T, Papoutsi C. Studying complexity in health services research: desperately seeking an overdue paradigm shift. BMC Med. 2018;16(1):95.
8. Callaghan G. Evaluation and negotiated order: developing the application of complexity theory. Evaluation. 2008;14(4):399-411.

For peer review only - http://bmjopen.bmj.com/site/about/guidelines.xhtml

9. Kernick D. Wanted—new methodologies for health service research. Is complexity theory the answer? Fam Pract. 2006;23(3):385-90.
10. Thompson DS, Fazio X, Kustra E, et al. Scoping review of complexity theory in health services research. BMC Health Services Research. 2016;16(1):87.
11. Clark AM. What are the components of complex interventions in healthcare? Theorizing approaches to parts, powers and the whole intervention. Social Science & Medicine. 2013;93:185-93.
12. Braithwaite J, Churruca K, Long JC, et al. When complexity science meets implementation science: a theoretical and empirical analysis of systems change. BMC Med. 2018;16(1):63.
13. Caffrey L, Wolfe C, McKevitt C. Embedding research in health systems: lessons from complexity theory. Health Res Policy Syst. 2016;14(1):54.
14. Chandler J, Rycroft-Malone J, Hawkes C, et al. Application of simplified Complexity Theory concepts for healthcare social systems to explain the implementation of evidence into practice. J Adv Nurs. 2016;72(2):461-80.
15. Trimmer JD. Response of Physical Systems. New York: Wiley & Sons; 1950.
16. Hawe P. Lessons from complex interventions to improve health. Annual Review of Public Health. 2015;36(1):307-23.
17. May CR, Johnson M, Finch T. Implementation, context and complexity. Implementation Science. 2016;11(1):141.
18. Hawe P, Shiell A, Riley T. Complex interventions: How "out of control" can a randomised controlled trial be? British Medical Journal. 2004;328:1561-3.
19. Byrne D. What Is complexity science? Thinking as a realist about measurement and cities and arguing for natural history. Emergence. 2001;3(1):61-76.
20. Clare L, Bayer A, Burns A, et al. Goal-oriented cognitive rehabilitation in early-stage dementia: study protocol for a multi-centre single-blind randomised controlled trial (GREAT). Trials. 2013;14(1):152.
21. Kudlicka A, Clare L. Cognitive rehabilitation in mild and moderate dementia. 2018. In: Oxford Research Encylopedia of Psychology [Internet].
22. Clare L, Kudlicka A. Goal-Oriented Cognitive Rehabilitation in Early-Stage Alzheimer's and related Dementias: Multi-Centre Single-Blind Randomised Controlled Trial (GREAT): Handbook for Therapists. Bangor: University of Bangor; 2015.
23. Warmoth K, Morgan-Trimmer S, Kudlicka A, et al. Reflections on a personalized cognitive rehabilitation intervention: Experiences of people living with dementia and their carers participating in the GREAT trial. Neuropsychological Rehabilitation. 2020:1-19.
24. Braun V, Clarke V. Thematic analysis. In: Cooper H, editor. APA Handbook of Research Methods in Psychology 2012. p. 57-71.
25. Ritchie J, Spencer L, O'Connor W. Carrying out qualitative analysis. In: Ritchie J, Lewis J, editors. Qualitative research practice: a guide for social science students and researchers. London, UK: SAGE Publications; 2003. p. 219-62.
26. Braun V, Clarke V. Using thematic analysis in psychology. Qual Res Psychol. 2006;3(2):77-101.
27. Maxwell JA. A Realist Approach for Qualitative Research. London: Sage; 2012.
28. Mitchell G, Agnelli J. Person-centred care for people with dementia: Kitwood reconsidered. Nursing Standard. 2015;30(7):46-50.
29. Delgado C, Upton D, Ranse K, et al. Nurses' resilience and the emotional labour of nursing work: An integrative review of empirical literature. International Journal of Nursing Studies. 2017;70:71-88.
30. Toomey E, Hardeman W, Hankonen N, et al. Focusing on fidelity: narrative review and recommendations for improving intervention fidelity within trials of health behaviour change interventions. Health Psychology and Behavioral Medicine. 2020;8(1):132-51.

For peer review only - http://bmjopen.bmj.com/site/about/guidelines.xhtml

31. Solman B, Clouston T. Occupational therapy and the therapeutic use of self. British Journal of Occupational Therapy;79(8):514-6.
32. Rahkonen T, Eloniemi-Sulkava U, Paanila S, et al. Systematic Intervention for Supporting Community Care of Elderly People After a Delirium Episode. Int Psychogeriatr. 2001;13(1):37-49.
33. Glanz K, Bishop DB. The role of behavioral science theory in development and implementation of public health interventions. Annual Review of Public Health. 2010;31(1):399-418.
34. Ling T. Evaluating complex and unfolding interventions in real time. Evaluation. 2012;18(1):79-91.
35. Gear C, Eppel E, Koziol-Mclain J. Advancing complexity theory as a qualitative research methodology. International Journal of Qualitative Methods. 2018;17(1).
36. Westhorp G. Using complexity-consistent theory for evaluating complex systems. Evaluation. 2012;18(4):405-20.
37. Grant RL, Hood R. Complex systems, explanation and policy: implications of the crisis of replication for public health research. Critical Public Health. 2017;27(5):525-32.
38. Brainard J, Hunter PR. Do complexity-informed health interventions work? A scoping review. Implementation Science. 2016;11(1):127.

For peer review only

For peer review only - http://bmjopen.bmj.com/site/about/guidelines.xhtml

Appendix 4: Methods and findings reported in accordance with the Standards for Reporting Qualitative Research (SRQR)

S1 Title	The title indicates the type of study and theoretical focus of the paper
S2 Abstract	An abstract with background, methodological detail and conclusions has been included
S3 Problem formulation	The introductory section comments on limitations of process evaluations and outlines how complexity theory could improve process evaluations
S4 Purpose or research question	The research question is stated in the first paragraph of the introduction.
Methods	
S5 Qualitative approach and research paradigm	The methods section states that the paper presents findings from a process evaluation embedded in a trial. The methods section describes the qualitative research as underpinned by a critical realist paradigm, and that qualitative interview and focus group data were analysed using a combined deductive and inductive approach.
S6 Researcher characteristics and reflexivity	The methods section states that the researchers analysing the data had no previous involvement in the study
S7 Context Setting/site and salient contextual factors	The introduction and background section provides information about the intervention setting, and includes references to more detailed publications about the study setting.
S8 Sampling strategy How and why research participants, documents, or events were	The methods section describes how participants were sampled. All available therapists were invited to a focus group, people with dementia were consecutively sampled across different sites, and therapy logs were sampled for around 10% of people receiving the intervention who had the best and worst outcomes from the intervention.

For peer review only - http://bmjopen.bmj.com/site/about/guidelines.xhtml

S9 Ethical issues	Details and reference number of the ethics approval are included
S10 Data collection methods	The timing and methods for data collection are described in detail in the methods section
S11 Data collection instruments	Data collection instruments are included in the appendices
S12 Units of study	Numbers and descriptions of participants are included
S13 Data processing	Transcription and use of NVivo are noted in the methods section. All data were stored on secure university servers.
S14 Data analysis	The method and stages of data analysis are described. The analysis of therapy logs drew on findings from the focus group – this is included in the description of methods.
S15 Techniques to enhance trustworthiness	The methods section states that focus group and interview data were collected by researchers without close involvement in the intervention. Data were analysed by researchers who had not been involved in the study up to that point (SMT and KW); developing themes from all datasets were discussed in meetings by the research team
Results/findings	
S16 Synthesis and interpretation	Findings are presented in relation to the theoretical concepts described in the introductory section.
S17 Links to empirical data	Quotes are included to substantiate the themes presented in the findings section
Discussion	
S18 Integration with prior work, implications, transferability, and contribution(s) to the field	Findings are summarised in the discussion section. Findings are also related to future implementation of the intervention and implications for the theoretical perspective (complexity theory)

For peer review only - http://bmjopen.bmj.com/site/about/guidelines.xhtml

S19 Limitations Trustworthiness and limitations of findings	Limitations of the study are included in the discussion section.
S20 Conflicts of interest	The authors have declared there are no conflicts of interest.
S21 Funding	The funder and grant number is stated.

For peer review only

For peer review only - http://bmjopen.bmj.com/site/about/guidelines.xhtml

Appendix 1: GREAT Focus Group Schedule - therapists' experience of providing goal-oriented cognitive rehabilitation for people with dementia (PwD) in GREAT

Introduction - aims of the focus group

The aim of this focus group is to explore therapists' experience of working with PwD to provide goal-oriented CR, to capture the nature of the interactions and the way in which the therapists deploy their skills to achieve the desired outcomes. We will use the results to inform the process of educating others on how to implement goal-oriented CR and to consider ways to further enhance CR in future.

- We value your open and honest responses. Please feel free to share your point of view even if it differs from what others have said. Every person's experiences and opinions are important.
- We are tape recording to capture everything you have to say, but we won't identify anyone by name in the report. The recording will be transcribed by someone from outside the team and anonymised.

General views on the process of providing goal-oriented cognitive rehabilitation – 10 min

How do you find the process of providing goal-oriented CR in GREAT?

What is it like to provide goal-oriented CR in GREAT?

Things that have worked well in providing goal-oriented CR – 10 min

What are the things that work well for you in the therapy process?

Which aspects do you look forward to most?

Challenges or difficulties in providing goal-oriented CR –10 min

What aspects of your work on providing goal-oriented CR in GREAT are most challenging?

What are the difficulties that you encounter when providing goal-oriented CR in GREAT?

Additional questions on the process of providing goal-oriented cognitive rehabilitation – 15 min

What sort of things have you learnt so far about working on cog rehab goals from working on the trial?

How do you find the role of the carer during the therapy process?

Has anything surprised you about the process?

Do you have hunches about what sort of people seem to benefit most?

If you were in charge, is there anything you'd be tempted to change about the way we have set up the study regarding working on the goals?

What do you think about the length of sessions?

What do you think about the overall length of the therapy?

How do you find training and supervision provided in GREAT?

If you were providing top tips to a therapist, what would be the key things you'd advise them to look out for or do?

Closing question

Before we finish the focus group, is there anything that we have missed? Is there anything anyone would like to say about their experience of providing goal-oriented CR in GREAT?

For peer review only - http://bmjopen.bmj.com/site/about/guidelines.xhtml

Summing up and moving on

Summarise main points of the discussion, balance negative comments with positive comments. Thank everyone for sharing their comments on providing goal-oriented CR in GREAT and end the focus group.

For peer review only - http://bmjopen.bmj.com/site/about/guidelines.xhtml

Appendix 2: Topics recorded by therapists in therapy logs

**Therapy log topic**	**Therapist ratings or comments recorded under relevant sessions**
Compliance	Whether given session was completed
Relationships	The relationship the therapist developed with the participant and carer, and the relationship between the participant and carer
Goals	Participant and carer responsiveness to the solution-focused problem-solving approach
	Goal 1: agreed strategies, between-session practice, and progress
	Goal 2: agreed strategies, between-session practice, and progress
	Goal 3: agreed strategies, between-session practice, and progress
	Any adjustments or modifications to goal statements provided at baseline
	In-session goal attainment ratings by participant, carer and therapist (sessions 10 and 14) and comments on these
	Selection of goal attainment scaling indicators for each goal (sessions 10 and 14) and comments on these
Activity levels	Review of activity levels, plan for behavioural activation to increase activity engagement, and comments on progress
Compensatory strategy use	Review of current use of compensatory strategies and environmental adaptations, plan to develop strategy use, and comments on progress
Restorative strategy use	Response to information about restorative strategies, plan for developing restorative strategy use, and comments on progress
Attention and concentration	Strategies introduced to help maintain attention and concentration, and progress with applying these strategies

For peer review only - http://bmjopen.bmj.com/site/about/guidelines.xhtml

Anxiety management	Current use of anxiety management strategies, carer's perspective on the participant's use of these strategies, introduction or refinement of anxiety management technique(s), and progress with use of anxiety management strategies
Carer well-being	Review with carer, and plan for enhancing carer well-being
Carer involvement	The extent to which the carer was engaged in supporting the process of therapy
Ending therapy	Plans for maintaining progress after the end of the intervention, and review of other sources of help and support
Experience of therapy	Review with the participant and with the carer, and therapist reflection on the process of therapy; therapist confidence in addressing participants' goals (following the nine month follow-up)

For peer review only

For peer review only - http://bmjopen.bmj.com/site/about/guidelines.xhtml

Appendix 3: Interview schedule for the person with dementia

The interview will take form of a conversation and the interviewer will encourage the participant to talk freely about the experience of the cognitive rehabilitation intervention.

The researcher will begin by re-establishing consent for the interview and for audio-recording.

The researcher may begin with some general conversation to build rapport as appropriate.

The researcher will introduce the main part of the interview by saying:
You've been taking part in the GREAT study and having visits from the therapist, and I'd like to know your views on what it was like. I'm interested in what it was like to take part in the study and how you found the visits from the therapist.

1. Experiences

The researcher will explore the participant's experiences and feelings starting with general questions:
How did you find the therapist's visits over the past few weeks?
What was it like to work with the therapist on your goals?

More specific information will then be elicited using prompts such as the following:
What were the more enjoyable things about your work with the therapist?
What were the less enjoyable things about your work with the therapist?
What aspects of your work with the therapist were more/less helpful?
Did you find it hard work to take part in the therapy?
What aspects of your work with the therapist were most challenging?
The interviewer will encourage the person to give specific examples, where possible.

2. Outcomes

The researcher will explore the impact of taking part in cognitive rehabilitation on the person's everyday life and self-perceptions.

What difference (if any) has your work with the therapist made to your daily life?
Has the experience changed anything in the way you think about your dementia/about yourself/about the future?
Has the experience changed anything in the way you relate to your carer/family?

The researcher will draw on positive comments from the participant to end the conversation on a positive note.

For peer review only

BMJ Open

Implementation processes in a cognitive rehabilitation intervention for people with dementia: A complexity-informed qualitative analysis

Journal:	*BMJ Open*
Manuscript ID	bmjopen-2021-051255.R1
Article Type:	Original research
Date Submitted by the Author:	19-Aug-2021
Complete List of Authors:	Morgan-Trimmer, Sarah; University of Exeter, College of Medicine and Health<br>Kudlicka, Aleksandra; University of Exeter, College of Medicine and Health<br>Warmoth, Krystal ; University of Hertfordshire, School of Health and Social Work & NIHR Applied Research Collaboration East of England<br>Leroi, Iracema; Global Brain Health Institute, School of Medicine<br>Oyebode, Jan; University of Bradford, Centre for Applied Dementia Studies<br>Pool, Jackie; QCS Quality Compliance Systems, Dementia Pal Ltd<br>Woods, Robert; Bangor University, Dementia Services Development Centre Wales, School of Health Sciences<br>Clare, Linda; University of Exeter, College of Medicine and Health & NIHR Applied Research Collaboration South-West Peninsula
**Primary Subject Heading**:	Health services research
Secondary Subject Heading:	Qualitative research
Keywords:	Dementia < NEUROLOGY, HEALTH SERVICES ADMINISTRATION & MANAGEMENT, QUALITATIVE RESEARCH

SCHOLARONE™
Manuscripts

I, the Submitting Author has the right to grant and does grant on behalf of all authors of the Work (as defined in the below author licence), an exclusive licence and/or a non-exclusive licence for contributions from authors who are: i) UK Crown employees; ii) where BMJ has agreed a CC-BY licence shall apply, and/or iii) in accordance with the terms applicable for US Federal Government officers or employees acting as part of their official duties; on a worldwide, perpetual, irrevocable, royalty-free basis to BMJ Publishing Group Ltd ("BMJ") its licensees and where the relevant Journal is co-owned by BMJ to the co-owners of the Journal, to publish the Work in this journal and any other BMJ products and to exploit all rights, as set out in our licence.

The Submitting Author accepts and understands that any supply made under these terms is made by BMJ to the Submitting Author unless you are acting as an employee on behalf of your employer or a postgraduate student of an affiliated institution which is paying any applicable article publishing charge ("APC") for Open Access articles. Where the Submitting Author wishes to make the Work available on an Open Access basis (and intends to pay the relevant APC), the terms of reuse of such Open Access shall be governed by a Creative Commons licence – details of these licences and which Creative Commons licence will apply to this Work are set out in our licence referred to above.

Other than as permitted in any relevant BMJ Author's Self Archiving Policies, I confirm this Work has not been accepted for publication elsewhere, is not being considered for publication elsewhere and does not duplicate material already published. I confirm all authors consent to publication of this Work and authorise the granting of this licence.

For peer review only

Implementation processes in a cognitive rehabilitation intervention for people with dementia: A complexity-informed qualitative analysis

Sarah Morgan-Trimmer, College of Medicine and Health, University of Exeter, St Lukes Campus, Heavitree Road, Exeter, EX1 2LU, UK. Email: s.morgan-trimmer@exeter.ac.uk (corresponding author)

Aleksandra Kudlicka, College of Medicine and Health, University of Exeter, Exeter, UK

Krystal Warmoth, Centre for Research in Public Health and Community Care (CRIPACC), School of Health and Social Work, University of Hertfordshire; NIHR Applied Research Collaboration East of England, UK

Iracema Leroi, Global Brain Health Institute, Trinity College Dublin, Dublin, Ireland

Jan Oyebode: Centre for Applied Dementia Studies, University of Bradford, UK

Jackie Pool, Dementia Pal Ltd, QCS Quality Compliance Systems, UK

Robert Woods, Dementia Services Development Centre Wales, School of Health Sciences, Bangor University, Bangor, UK

Linda Clare: College of Medicine and Health, University of Exeter, Exeter, UK; NIHR Applied Research Collaboration South-West Peninsula, UK

Wordcount: 6314

For peer review only

For peer review only - http://bmjopen.bmj.com/site/about/guidelines.xhtml

Abstract

Objectives: Health care is often delivered through complex interventions. Understanding how to implement these successfully is important for optimising services. This article demonstrates how the complexity theory concept of 'self-organisation' can inform implementation, drawing on a process evaluation within a randomised controlled trial of the GREAT intervention which compared a cognitive rehabilitation intervention for people with dementia with usual treatment.

Design: A process evaluation examined experiences of GREAT therapists and participants receiving the intervention, through thematic analysis of a focus group with therapists and interviews with participants and their carers. Therapy records of participants receiving the intervention were also analysed using adapted framework analysis. Analysis adopted a critical realist perspective and a deductive-inductive approach to identify patterns in how the intervention operated.

Setting: The GREAT intervention was delivered through home visits by therapists, in eight regions in the UK.

Participants: Six therapists took part in a focus group, interviews were conducted with 25 participants and 26 carers, and therapy logs for 50 participants were analysed.

Intervention: A 16-week cognitive rehabilitation programme for people with mild-to-moderate dementia.

Results: 'Self-organisation' of the intervention occurred through adaptations made by therapists. Adaptations included simplifying the intervention for people with greater cognitive impairment, and extending it to meet additional needs. Relational work by therapists produced an emergent outcome of 'social support'. Self-organised aspects of the intervention were less visible than formal components, but were important aspects of how it operated during the trial. This understanding can help to inform future implementation.

Conclusions: Researchers are increasingly adopting complexity theory to understand interventions. This study extends the application of complexity theory by demonstrating how 'self-organisation' was a useful concept for understanding aspects of the intervention that would have been missed by focusing on formal intervention components. Analysis of self-organisation could enhance future process evaluations and implementation studies.

Article Summary

Strengths and limitations of this study

- This study adopted an in-depth, theoretically-informed qualitative analysis to understand implementation processes in a complex intervention
- The final stage of analysis drew on three complementary datasets to develop a holistic understanding of how a complex intervention operated
- A limitation of the study is that it only reports therapist and participant perceptions of how the intervention operated; researcher observations of therapy sessions were not conducted.
- This study included a small number of focus group participants, interviewees and therapy records; the study was designed to produce theoretical generalisability through in-depth analysis rather than a large sample size.

For peer review only

Background

Introduction

This article discusses the application of the complexity theory concept of 'self-organisation' for understanding implementation. Implementation, defined here as *"the process through which interventions are delivered, and what is delivered in practice"* (1, pg. 8), is an important element of process evaluation. This is distinct from the definition of implementation within the field of 'implementation science' which *"is the scientific study of methods to promote the systematic uptake of research findings and other evidence-based practices into routine practice"* (2, pg. 1). While there is overlap between the two definitions, process evaluations tend to focus on '*the quality and quantity of what is actually delivered during the evaluation'* (1) and it is this focus we adopt in this article. It reports findings from a process evaluation which addressed several research questions, including "how was the intervention implemented?". Process evaluations may be the first opportunity to closely study, refine and optimise implementation of a new intervention. Findings on implementation can then inform wider roll-out of interventions at the post-trial stage (1).

Existing approaches to implementation employed by process evaluations face two main limitations. First, process evaluations are often structured using logic models which represent the underlying theory of how an intervention, including implementation processes, work (3). Logic models usefully provide a simple-enough model on which to base an evaluation, but in doing so they tend to conceptualise the world in a relatively linear, mechanistic way. Second, logic models focus attention on whether a certain process happened, rather than unexpected events, because they represent ideal versions of what researchers hypothesise will occur. Within this approach, implementation of the intervention tends to be examined in terms of fidelity to this ideal version. However, during implementation, some components of the intervention may not have been delivered as anticipated, additional components may have been introduced, and components may have interacted with each other or contextual factors in unexpected ways. These aspects may be missed if data are only collected on components identified in logic models. This article discusses the application of the complexity theory concept of 'self-organisation' for improving understanding of how complex health interventions are implemented. It examines how it can be applied through analysing process evaluation data from a trial of a community-based cognitive rehabilitation (CR) intervention for people with dementia (4, 5).

Complexity theory and self-organisation

Complexity theory has been increasingly advocated as an alternative theoretical lens for understanding complex interventions, and is being increasingly applied across a range of healthcare research areas and evaluation studies (6-12). It theorises systems of different types – social, physical, biological – as constantly self-organising and unfolding. A complex system cannot be understood only in terms of its parts, as a complicated machine for example, where the causal powers of the system are explained by its components (6, 9, 13). Processes occur in complex systems through the dynamic, non-linear relations between components at different levels as interacting parts of a system, producing effects in unpredictable ways (14-16). Complexity theory encompasses a range of concepts which describe how patterns occur, including self-organisation, feedback loops and

For peer review only - http://bmjopen.bmj.com/site/about/guidelines.xhtml

emergence (10, 14, 15, 17). Complex health interventions can be conceptualised as complex systems since they incorporate multiple interacting elements such as humans (as active and responsive agents), organisations, materials, rules, policies and so on, through which these patterns may occur (6, 13, 14). Complexity approaches also conceptualise the system within which an intervention is introduced as complex, unfolding, interacting with and changed by the intervention: systems are nested within other systems and cannot be isolated (6, 15, 17-19).

This article draws on the complexity concept of 'self-organisation', and the additional concepts of 'emergence' and 'feedback loops', and explores their application to studying implementation. 'Self-organisation' refers to interacting elements of a system, such as practitioners, who behave or make individual micro-level decisions in response to other elements of a system (such as clients) in a way which produces patterns or order at a higher level (10, 15). This order is produced in a bottom-up way since it occurs as the result of individual decisions which are not coordinated, rather than through top-down instructions. The order which is created is 'emergent', as it appears at the overall intervention level and occurs somewhat unpredictably. A common form of self-organisation occurs through feedback loops, for example where practitioners tailor how they deliver an intervention in response to client reactions to the intervention (13).

Self-organisation can operate within or beyond the formal design of an intervention. Some interventions are designed to be partly self-organising, where the practitioner is expected to adapt the intervention according to individual client needs for example, as long as the core functions of the intervention are delivered (20, 21). The core functions relate to ways in which the intervention activities produce mechanisms of action, and must be retained for an intervention to be effective, while the activities (form) of an intervention may be adapted (22). Other forms of self-organisation occur outside of the prescribed parameters of the intervention, through deviation from protocols for example. Through micro-level decisions about adaptations, practitioner behaviours result in self-organisation as their patterns of behaviour at a collective level shape what intervention is actually delivered. Self-organisation is not commonly theorised as being part of implementation processes, yet could significantly influence implementation and, in turn, intervention outcomes.

While complexity theory may explain the real world more adequately than linear models, there are challenges in applying it within a process evaluation. Complexity theory is a collection of related concepts which are often presented in overlapping rather than standardised lists, and does not have an agreed definition (10). It is not yet a consolidated theory in that there are questions about how concepts are related to each other (9, 17). Complexity theory also tends to emphasise the unpredictability of outcomes and produce post-hoc explanations rather than having strong predictive power (10, 23, 24). This is somewhat at odds with the aim of process evaluation, and research more generally, to identify generalisable patterns, 'demi-regularities' or predictive theories (6). However, the emphasis on unpredictability varies within the family of complexity theory approaches (6). This article examines the application of complexity concepts, particularly 'self-organisation', in a process evaluation and reflects on its use for understanding the implementation of complex health interventions.

The paper draws on several datasets which answered a set of related questions for the process evaluation about how the intervention operated. The research questions were: how was the

For peer review only - http://bmjopen.bmj.com/site/about/guidelines.xhtml

intervention delivered; what was the feasibility of delivering the intervention; was the intervention delivered with fidelity; what were the mechanisms of impact; what influenced treatment outcomes; what were participant experiences of the intervention; and what were therapists' experiences of delivering the intervention? The analysis reported here answered a further research question: how did the way the intervention was delivered influence whether and how participants benefitted? The broad aim of a process evaluation is to explain how an intervention operates to produce its outcomes, but process evaluations often only address different elements of an intervention separately. This approach, drawing on complexity concepts, was an attempt to conduct a more holistic analysis.

The GREAT trial

The GREAT study was a randomised controlled trial, with an embedded process evaluation, of a CR intervention for people with a clinical diagnosis of dementia (referred to here as 'participants') and a Mini Mental State Examination (MMSE) score of ≥ 18 points indicating mild to moderate cognitive impairment (25, 26). It was delivered in eight regions in the UK. The main purpose of the intervention was to improve ability to carry out everyday activities in the areas the participant chose to target. The intervention involved identifying with the participant personally relevant and significant goals related to daily activities, and then working together to develop and implement a set of strategies to enable the person to achieve the desired outcomes. The primary outcome was participant-reported progress towards participant-identified goals at three months. It was delivered by a practitioner (eight of whom were occupational therapists and one a nurse) during one-hour home visits, ideally with a carer also present at part of the session. The participant, with carer support, was encouraged to implement agreed changes in daily routines and practise strategies between visits. Ten visits were delivered over three months, followed by four maintenance sessions over six months. The intervention was detailed in a practitioner handbook which included a structured protocol (27); the core component of sessions was to work towards personalised goals, supplemented by strategies to improve attention and concentration, compensatory strategies, and restorative strategies for retaining new information or improving recall. Additional optional components included anxiety management, increasing activity levels, discussing carer well-being, and signposting to other services. Training sessions and regular group and individual supervision was provided for the therapists. The intervention is conceptualised here as a complex intervention as it had multiple interacting components, addressed difficulties encountered in dementia for both participants and carers, was a tailored intervention and targeted multiple outcomes (28). Full details of the intervention, trial methods and trial outcomes have been published (4, 5, 25, 29).

The intervention was personalised in that participants could identify up to three goals they wished to work towards and the therapists applied CR strategies to address these. The intervention was also designed to be responsive to participant needs in that therapists had some flexibility to apply elements of the intervention such as anxiety management, depending on the needs of the participant and their personal and social contexts. The handbook reflected this balance between structure and flexibility, for example:

For peer review only - http://bmjopen.bmj.com/site/about/guidelines.xhtml

The intervention in a trial needs to follow a structured protocol, as summarised in Table 1 below. However, some flexibility will be needed as participants will have varying needs and preferences and will progress at different rates.
GREAT Handbook for Therapists (27: 40)

This article examines the implementation of the GREAT intervention, where the intervention is conceptualised as a complex intervention designed to incorporate a degree of self-organisation through its person-centred design and requirement to be responsive to the contexts, needs and preferences of people with dementia.

Methods

This article draws on findings from three datasets which were analysed for the GREAT trial process evaluation(4). Methods and findings are reported in accordance with the Standards for Reporting Qualitative Research (SRQR) checklist (Appendix 1). A focus group and analysis of therapy logs from the intervention examined the perceptions and experiences of therapists about how the intervention was delivered, feasibility of the intervention, fidelity to the intervention protocol, and perceived factors affecting treatment outcomes. A set of interviews explored the perceptions and experiences of the intervention by participants and their carers and whether and how any impact from the intervention was experienced by this group. Written informed consent was obtained at the beginning of the trial. Trial researchers and therapists were trained to monitor ongoing consent and identify and respond to any indication of possible withdrawal of consent. Researchers began participant and carer interviews by re-establishing consent. A critical realist perspective was adopted in the data analysis to identify causal patterns of how the intervention operated (a realist ontology) while allowing that perceptions of the intervention may differ between participants (a relativist epistemology). To this end, the three datasets were initially analysed with an overall focus on participant perspectives, whether these were categorised according to deductive categories identified from previous research or inductively developed. Additional interpretive work was conducted using a graphic to relate themes from the focus group analysis to each other, to draw out a more realist explanation of the dynamic relationships between different elements of the intervention. In a second stage, a realist-oriented analysis of how the intervention operated was conducted though deductively applying constructs from the complexity literature to the initial findings from the three datasets.

Focus Group

A focus group was conducted with all six therapists who were in post at the time, at the end of the first year of the intervention, to examine their experiences of the intervention (see Appendix 2 for schedule). The focus group was conducted as part of the second annual training event for the trial therapists. The discussion was facilitated by co-author and trial co-investigator JO. She had a thorough understanding of the intervention but was not involved in supervising the therapists, which facilitated an open discussion. It was digitally recorded, transcribed verbatim and uploaded to NVivo 11. A thematic analysis, underpinned by a critical realist perspective, was conducted by the lead author, SMT, who had not been involved in the study up to this point (30). Data were initially

For peer review only - http://bmjopen.bmj.com/site/about/guidelines.xhtml

coded using a combined inductive-deductive approach, drawing on the dementia literature informing the GREAT trial (4). Themes were then developed, after first summarising the codes, and graphics were developed to represent and analyse themes at a more interpretive level. This article draws on three themes relevant to implementation: the perceived influence of the severity of dementia; adaptation work of therapists in response to their perceptions of the severity of dementia; and the relational work implemented by therapists and the outcomes this produced.These themes are more fully described in the findings section.

Analysis of therapy logs

Therapy logs were maintained for every participant for each of the 14 sessions and included detail about each intervention component, such as progress towards goals (see Appendix 3 for categories included in therapy logs). These logs were analysed by SMT to try to identify factors which might explain how participants benefitted from the intervention, or not. Therapy logs were analysed to compare the 25 participants with the highest-score primary outcomes (the 'good outcomes' group) and the 25 participants with the lowest-score primary outcomes (the 'poor outcomes' group), out of the intervention arm population of 281 individuals. The primary outcome was participant-reported goal attainment at 3 months post-randomisation, measured using the Bangor Goal-Setting Interview (BGSI) (4). This measurement was undertaken through a home visit by a researcher who was blinded to the trial arm of the participant.

An adapted framework analysis method was employed, using deductive categories identified in the focus group findings and also inductive categories where novel factors were identified (31). The analysis aimed to identify differences in patterns in each group, paying attention to both qualitative and quantitative differences. First, each component for each session was summarised and compared between the 'best outcomes' and 'poor outcomes' groups. Second, the trajectory of each participant's progress through the intervention was summarised and then compared by group. Third, a 'negative case analysis' was conducted to explore factors which did not fit with the general patterns emerging from the first two stages of the analysis.

Interviews

Face-to-face, semi-structured interviews were conducted with 25 participants and 26 carers at nine months post-intervention, to explore their experiences of the intervention (see Appendix 4 for interview schedule). They were consecutively sampled across three different sites sites (North Wales, South Wales and Greater Manchester) where the intervention was taking place and where there was a research assistant not otherwise involved in the trial available to complete the interviews. Participants and carers were interviewed separately wherever possible, starting with the person with dementia. Interviewers took a photograph of the therapist on the visit to prompt the participant's memory of the therapy sessions. If the participant was struggling to recall the therapy sessions, the interview was completed jointly with the carer. All interviews were audio-recorded, transcribed verbatim and uploaded to NVivo 11 (4).

Data were analysed using thematic analysis which was underpinned by a critical realist position and which employed an inductive approach to identifying and exploring patterns of meaning (32, 33). Data were initially coded by four researchers, and then organised into meaningful groups by KW and SMT. Related themes were clustered together and organised into an overall thematic map.

For peer review only - http://bmjopen.bmj.com/site/about/guidelines.xhtml

Detailed methods and findings of the focus group, therapy log analysis and interviews have been reported (4). In a last stage of analysis, themes from the focus group analysis were interpreted in relation to core concepts in the complexity literature. The core concepts considered were: interacting elements, unpredictability, self-organisation, emergence, non-linearity, fuzzy boundaries, feedback loops and being 'more than the sum of its parts' (9, 10, 14, 15, 17). These themes were then further developed through triangulation with findings from the interviews and therapy log analysis. Findings on intervention implementation are reported below.

Results

Findings presented here explain how the intervention operated as a complex system, and how some aspects of implementation occurred in a self-organising pattern, through adaptation behaviours of therapists. These were: simplifying the intervention for people with greater cognitive impairment; providing additional support in response to participant needs; and conducting relational work to engage participants, which produced an emergent outcome of increased social support.

Self-organisation through adaptive response

The GREAT intervention incorporated self-organisation in its design: therapists were required to select CR strategies appropriate for individuals and their social context, and tailor the intervention to individuals' needs and preferences. Participants and carers reported experiencing the intervention as personalised:

> *[Goals] were always relevant to … obviously relevant to the issues that [therapist] wanted to raise … And also relevant to, the issues that were important for [person with dementia] … she worked at a pace that was good for him as well.*
> (Interview, Carer 5)

Therapists reported that they adapted the intervention in response to their perception that participants with greater cognitive impairment (within the trial population range) were less likely to engage in and benefit from the intervention. For example, therapists observed that these participants had more difficulty setting relevant goals or remembering them. Therapists' notes for participants in the 'poor outcomes' group also described features which could be attributable to greater cognitive impairment: a tendency to set more basic-level goals; being more likely to give up on a goal; lower levels of motivation; being more withdrawn during sessions; and having less awareness regarding their condition.

Therapists responded to participants with greater cognitive impairment through adapting their delivery of the intervention for these participants in several ways. First, because it was difficult for these participants to absorb information, therapists would slow down the pace of delivery and only deliver what they thought were the most relevant sections:

> *Therapist 5: I was almost going at a snail's pace, because I realised it was far too much information to dish out, and I was literally cherry-picking the bits I thought were relevant so I*

For peer review only - http://bmjopen.bmj.com/site/about/guidelines.xhtml

could get through the goals and get them rated, and just literally [Several people agreeing]
Therapist 4: And, sometimes you do have to cherry pick don't you? [Several people agreeing].
(Therapist focus group)

Therapists also tailored the timing of material, moving some supplementary topics to an alternative session for example:

Therapist 6: But there's too much information for me, so let alone someone who has to take a while, or so I've started moving things away
Therapist 4: Oh, I've moved things very early on, I moved the anxiety stuff, unless it's the glaring, big problem, I move anxiety from week 1 to week 2 because it's too much information.
(Therapist focus group)

In addition, therapists reported that they simplified some of the language in the handbook:

Therapist 7: The 'restorative' and 'compensatory', that's too jargon-y, and too heavy for the person, not all, but some of the people with dementia…
Therapist 2: Yes, I've had to change the words to 'methods', because 'strategies' just scares them.
(Therapist focus group)

In these ways, therapists made individual, micro-level decisions to ensure the intervention matched the needs of participants with greater cognitive impairment, and sometimes adapted the content from the intervention protocol (27). This is an example of self-organisation operating in a feedback loop, where practitioners adapted an intervention according to the perceived capacity of people with dementia. Through this adaptation work, they shaped the nature of intervention for some participants.

Self-organisation through extending the intervention

Carers were important for the delivery of the intervention because they supported the practice of new techniques, and could also impact on participant motivation. Difficulty engaging some carers was noted in the therapy logs and, in the focus group, therapists reported additional efforts they made to engage carers. For example, therapists sometimes adjusted the time allocated (often increasing it) for home visits and also changed the timing of visits to ensure they met with carers:

Therapist 7: I mean I've done home visits at 8 o'clock in the evening, just to catch up with the carer, so I can have face-to-face and actually get what's going on, because I can't get hold of her during the day, she just doesn't answer her phone. So, having to do home visits in the evening, so that's impacting, that's where the hours are coming in, from doing more of that work…
Therapist 6: I've often gone out on home visits, and they've [the carer has] gone out. Then you have to extend your visit by an extra 20, 30 minutes because, you don't want to just leave… So you have to extend your visit sometimes, or you have to ring [the carer] afterwards.
(Therapist focus group)

For peer review only - http://bmjopen.bmj.com/site/about/guidelines.xhtml

Therapists sometimes also had to take additional time to identify and meet additional carers who were most likely to be the person supporting the intervention but not the carer who was originally nominated to take part in the intervention:

> *Therapist 4: Actually the workload increases for us, because what we're doing is we're talking to the carer that's on the spot, and we're also having to liaise with the son and daughter, either by text or email, or something else in the evenings, that has quite a massive implication [agreement from others].*
> (Therapist focus group)

Therapists also delivered several 'add-on' components in response to the needs of participants. First, the intervention component to address carers' levels of well-being was formally limited in the handbook to referring carers to local sources of support: *You can direct carers to appropriate sources of support in the local area, and encourage them to access these (27: pg. 51)*. However, one therapist reported contacting services directly:

> *Therapist 7: I'm also contacting social services, so that the carer will get a break, that shouldn't be part of my role, but no-one else seems to.*
> (Therapist focus group)

Second, therapists commented on (often marital) conflict in the relationships between participants and their carers, also noted in therapy logs, and attempted to reduce conflict even though they were aware it was beyond the parameters of the intervention:

> *Therapist 2: I do find that it's about the nature of the relationship as well. And often you do find, like you're doing a couples intervention, it's not just about the dementia, it's often about the dynamics that have probably gone on through their whole relationship but the situation is highlighting it, and that's really quite difficult to manage, isn't it?*
> *Therapist 6: Sometimes it's like marriage counselling*
> *Therapist 2: Yeah… it's about their relationship, isn't it, it is a bit of the couples stuff, and I find myself doing that, and I think it is helpful, but it's beyond what we've been given.*
> (Therapist focus group)

Several carers described in interviews how the therapist acted as a mediator in conflicts between the carer and person with dementia. Carers also reported improved relationships between themselves and participants as a result of the intervention, for example having increased understanding of and patience with the person with dementia. Several participants commented that they had greater social awareness and were more likely to consider the impact of what they said on others.

In these ways, self-organisation occurred through therapists' micro-level decisions to adjust (often increase) the time taken to engage carers, directly contact services, and reduce conflict between carers and people with dementia. Some therapists extended the intervention so that it was larger in scope than described in the handbook. These patterns of self-organisation can also be understood in

For peer review only - http://bmjopen.bmj.com/site/about/guidelines.xhtml

term of feedback loops, where therapists' adaptive behaviours were in response to the contexts and unmet needs of people with dementia and their carers.

Self-organisation in relational work and emergent 'social support' outcomes

Therapists engaged in building relationships with participants and their carers; the intervention handbook described one of the therapist's roles as '*Developing rapport with participants and carers and building good relationships' (27: pg. 42)*. In interviews, people with dementia described positive relationships with therapists:

> *Oh fine, yeah fine, got on well … Easy, yeah she explained everything and, you know, it was no hardship (laughs)…That's right, yeah, well sometimes when people come to see you, … you're afraid to talk, you know, afraid to say anything when it's a little bit dumb. But she made me feel so comfortable and within a couple of minutes we were just like as though we'd been friends for a long time.*
> (Interview, Person with dementia 1)

Therapists reported that relationships with participants were an important aspect of the intervention, helping to engage people with dementia in the intervention and motivate them:

> *Therapist 2: The actual nature of the relationship and that therapeutic rapport, which I think actually counts for a lot, but I think it largely goes unmeasured in a way, what we bring as people, and our relationship, and that's the motivating factor, and it's hard to know how you would measure that, but I think there is a lot about them getting to know you and you becoming part of their routine, and that goes a long way*
> *Therapist 5: I think they open up don't they*
> (Therapist focus group)

A good relationship was also important for therapists' work in that getting to know participants well underpinned their ability to help participants identify relevant goals and develop personalised strategies.

Additionally, therapists recognised that their relational work and the provision of social support as being important in itself and would dedicate time specifically to this aspect of their visits:

> *Therapist 6: I think sometimes it's that really good for the carer and the participant to have somebody going in and for such a long time. You do work on the goals, but another big part of it is quite, you know, supportive, and you know, social, because you spend the first ten minutes of your visit, they just go on about what they've done in the past week, and you can't be like, come on let's crack on [laughter] I think you do see in a lot of people, especially as you go on, they thaw a bit as you visit and then [several people agreeing] I get that good bond by session ten, and you're sad to pull out, but I think that support does really help.*
> (Therapist focus group)

For peer review only - http://bmjopen.bmj.com/site/about/guidelines.xhtml

This relational work was self-organising in that therapists conducted relational work and provided social support, going slightly beyond the intervention parameters by regarding it as an intervention component in itself and spending more time on this than reflected in the handbook.

The social support provided by the therapists also developed into an emergent outcome of the intervention in that it was perceived as the main benefit for some participants:

> *Therapist 7: the pleasure of seeing people try and do well, even if they're not achieving their goals, it's the other things that they're getting from it, the social interaction, the time to talk about their condition, the dementia, and it not being hushed away and in the cupboard.*
> (Therapist focus group)

In interviews, some people with dementia were unable to recall the goals they had been working towards but many commented on the relationship with the therapist and that they would miss the visits now the therapy had ended. Carers also commented on relational benefits for people with dementia:

> *I think my mum just enjoyed it more that somebody was, the social aspects of it, that somebody was coming*
> (Interview, Carer 1)

The therapy log analysis indicated that relational and social support outcomes were an important (or at least the best-recalled) element of the intervention by people with greater cognitive impairment. At the end of the intervention, participants from the 'poor outcomes' group were more likely to refer to the relational or social aspect of the therapist visiting them as a positive element of the intervention, whereas participants in the 'best outcomes' group were more likely to give examples of formal components of the intervention that had benefitted them. One therapist also commented in the focus group that social support could be the primary benefit for the carer.

In these ways, relational work conducted by therapists and their provision of social support extended the scope of intervention as it was described in the handbook. Self-organisation occurred through micro-level decisions of therapists which helped them deliver the intervention successfully but also meet the social support needs of participants and in doing so developed into an emergent outcome.

Discussion

This article provides an example of how process evaluation findings can be interpreted using complexity theory and the concept of self-organisation. Some implementation processes occurred as self-organisation, through individual decisions made by therapists. These decisions produced 'order' at a higher level by creating patterns of service delivery and outcomes. The self-organisation of the intervention occurred partly by design, through a person-centred approach, but also through adaptions (to severity of dementia), extensions (of therapists' time and social support) and add-on components (carer support and relationship conflict resolution) (34). The handbook recommended but did not include detail about the adaptation of the intervention, and also implied flexibility in

For peer review only - http://bmjopen.bmj.com/site/about/guidelines.xhtml

relation to the delivery of formal components rather than extending the time allocation or scope of the intervention (27). It was not always clear, therefore, where self-organisation fell within or outside of the intervention parameters. Some of the adaptations resulted from therapists' interpretation of the flexibility allowed within the intervention as an aspect of its personalised approach, to achieve participants' goals which was the core component of the intervention. Therapists are likely to have drawn on their professional training and experience to make decisions about when and how to adapt the intervention. For example, skilled therapists such as OTs would generally translate complex material for use with clients, such as simplifying language in a handbook. Therapists also felt they were going beyond the intervention design at times, particularly in feedback loops when they provided 'add-ons' in response to participants' needs and contexts such as addressing relationship conflicts. Implementation within a trial context meant the intervention was described in a structured way in the handbook and there was less flexibility than would be the case in normal clinical practice. This may have led therapists to view the intervention as relatively structured and been more likely to view their adaptations as falling outside the intervention parameters.

The findings about self-organisation have several implications for further studies or wider roll-out of the GREAT intervention. First, the adaptations made by therapists for people with greater cognitive impairment (within the trial range of a Mini Mental State Examination score of ≥ 18 points) could be added to the handbook as examples of tailoring to the specific needs of the person, and inform training for therapists. Second, referrals to other services and specialised professionals could be enhanced in order to meet the wider needs of people with dementia and their carers, particularly for common issues such as relationship conflict. Third, social support needs could be addressed by referral to an alternative, less resource-intensive intervention such as befriending. Alternatively, the emergent outcome of social support, particularly for those with greater cognitive impairment, could be more formally incorporated into the intervention design. This may require increased resourcing, including formally incorporating this skill into recruitment and training for professionals, depending on what prior training practitioners have received (35). This intervention was delivered mainly by skilled OT therapists who typically offer a range of types of support; if the intervention was delivered by OT assistants, for example, additional training might be required. This also depends on whether future interventions are defined as targeted CR interventions or expanded to incorporate the additional elements observed in this study. Fourth, the time allocation for visits could be revised, or made more flexible, to accommodate the additional time requirements for some visits due to the extended or 'add-on' components provided. Lastly, future evaluations of the intervention could investigate the impact of social support for participants and carers, since findings indicated this area was important but an in-depth examination of this was beyond the scope of the study.

This analysis had several limitations. First, the qualitative data are the views and perceptions of the therapists, participants and carers of how the intervention operated, and the therapy logs were relatively brief notes made by therapists. Second, only one focus group of six therapists was conducted, and the therapy log analysis was limited to 50 participants. However, this analysis was designed to produce theoretical generalisability through in-depth analysis rather than a large sample size.

For peer review only - http://bmjopen.bmj.com/site/about/guidelines.xhtml

Applying a complexity theory lens had advantages over traditional process evaluation approaches. It explained how decisions dispersed throughout a system at the micro-level interacted with population characteristics and context, and how this influenced what intervention was delivered and what outcomes it produced. It went beyond describing practitioner adaptations, as it showed how adaptation can create 'order' in general patterns of behaviour and in creating outcomes other than that which was originally intended by the intervention (in this case, social support). The idea of self-organisation also specifically conceptualises decisions made by practitioners as a 'bottom up' phenomenon which emphasises the less predictable nature of this kind of behaviour. This is in contrast to the fidelity and adaptation literature which emphasises defining core form and function, and peripheral aspects of interventions, to identify what should be planned or controlled (22, 36, 37). The type of self-organising feedback loops described here is likely to be a common pattern in health interventions, where practitioners adapt interventions to meet the needs of their clients. It may be particularly common where participants have high levels of unmet needs, co-morbidities or in complex cases. Future studies of implementation could analyse the micro-processes of self-organisation specifically, in addition to formal components of interventions. This type of approach requires an agnostic position on fidelity of intervention delivery since lack of adherence to intervention protocols is not necessarily a negative aspect of the intervention (38). Although some degree of fidelity is important for studies such as trials, understanding how adaptation may occur is important for real-world implementation. Adaptive behaviours could be positive in that they support individuals, help engage participants, and help tailor the intervention to a local context (14, 39, 40). However, adaptive behaviours could also create difficulties if they expand the scope of the intervention beyond what is possible for sustained delivery, or are unsuccessful. Expansion could displace the delivery of core components of the intervention, for example, or lead to burnout in practitioners. This depends partly on how the intervention is being developed and refined during process evaluations: in some early-stage studies, adaptations by practitioners in practice may be helpful. However, when testing a well-defined intervention in a definitive trial, adaptations could be more problematic. Clarity about the balance required between fidelity and adaptation, and the trade-offs involved, is therefore necessary (41).

A complexity perspective was also useful in that it identified informal, less visible processes which might not be picked up by standard evaluation models measuring fidelity against formal components and protocols. One particularly informal and non-visible aspect of the intervention was the relational work conducted by therapists and the provision of social support through this. Findings from this study were that it was an important aspect of how the intervention operated: it facilitated participant and carer engagement in the intervention, supported aspects such as personalisation, and underpinned improved social support valued by participants and their carers. While relational and social support work may be recognised by practitioners as a common micro-level process, it is given comparatively little attention in published health intervention research (42, 43). This study suggests it is worthy of more attention in health intervention evaluation studies.

Complexity theory was used here to retrospectively explain the implementation of an intervention, which is less useful than predictive theory. However, the latter is challenging (10, 44). Rather than being predictive in a strong sense, complexity theory could inform programme theories and logic models to include commonly-occurring patterns such as self-organisation or feedback loops (45).

For peer review only - http://bmjopen.bmj.com/site/about/guidelines.xhtml

Complexity constructs could be used deductively in qualitative data analysis, for example. In this study, they were particularly useful in the later stages of analysis when findings were considered together to try to understand how the different elements of the intervention worked together. This could be supported through developing less linear logic models and applying them flexibly as programme theory develops during an evaluation (3, 19). Emergent outcomes could be considered as one potential outcome in a logic model, in addition to pre-specified primary and secondary outcomes. Adaptation and feedback loops could also be incorporated in logic models, as suggested in the MRC process evaluation guidance (1). To this end, complexity theory could be more useful for process evaluation as a methodological theory to guide modelling, data collection and analysis, or an overarching framework, rather than a type of theory that explains how the world works in a strongly predictive sense (9, 46). An alternative approach is to combine complexity approaches with other theoretical perspectives which explain agency or structures at different levels of a system (6, 8, 47). Both approaches require multiple, exploratory and flexible methods, including qualitative methods of sufficient depth, in order to identify informal, complex and unpredictable patterns (6, 7, 12, 40, 48). While complexity theory is increasingly employed, it is still a relatively new approach in complex health interventions research, particularly in empirical research (40, 49). Calls have been made for further examples of its application as well as better operationalisation of its concepts (1, 7, 49, 50). This article has provided one example of how complexity theory, particularly the concept of self-organisation, can be useful for providing insight into the implementation of an intervention that would have been missed by a process evaluation only focusing on formal intervention components.

Author contributions

SMT designed and conducted the analysis of the focus group and therapy log data, oversaw the analysis and discussed themes from the interview data; and devised and drafted the paper; AK discussed themes developed from the focus group and therapy log data, and also commented on several drafts of the paper; KW analysed interview data and commented on several drafts of the paper; JO conducted the focus group, contributed to the study design and commented on several drafts of the paper; Il, JP, and RW contributed to the study design and commented on several drafts of the paper; LC oversaw the study design, discussed themes developed, and commented on several drafts of the paper.

Disclaimer

KW is now supported by the NIHR Applied Research Collaboration East of England. The views and opinions expressed therein are those of the authors and do not necessarily reflect those of the HTA programme, Applied Research Collaboration South West Peninsula, Applied Research Collaboration East of England, NIHR, NHS or the Department of Health and Social Care.

Acknowledgements

We thank all the participants with dementia and their family supporters who generously contributed their time to be part of this study. We also thank our experts by experience and staff at Alzheimer's Society for supporting the study. We would like to acknowledge the key roles played by Ho Yin Chan, Matthew Lewis and Julie Nixon who conducted the qualitative interviews, Dr Gill Toms who

For peer review only - http://bmjopen.bmj.com/site/about/guidelines.xhtml

conducted some of the qualitative data analysis, and the trial therapists who provided the intervention.

Patient and Public Involvement

For the GREAT trial, in which the process evaluation was embedded, experts by experience including Alzheimer's Society Research Volunteers were consulted at the set-up stage to inform participant information resources and trial procedures. As a result we made a number of amendments to the participant-facing documents and assessment measures. The inclusion of qualitative interviews and a focus group in the study design was at the suggestion of the experts by experience. They also provided insightful comments about the progress of the trial and contributed to developing a follow-up application for an implementation grant. Study participants were updated about trial progress through a regular newsletter.

Trial registration

ISRCTN21027481

Ethics approval statement

The study was reviewed by Wales Rec 5, which issued a favourable opinion on 25th June 2012 (Reference 12/WA/0185), and was approved by the Bangor University School of Psychology Research Ethics Committee.

Reporting statement

Methods and findings are reported in accordance with the Standards for Reporting Qualitative Research (SRQR) checklist (see appendix 1).

Competing interests statement

The authors declare they have no competing interests.

Funding statement

This work was supported by NIHR HTA, grant number 11/15/04.

Data sharing statement

Interview and focus group data were not deposited in an archive due to the small numbers of individuals participating in named sites, which could compromise anonymity through potentially identifiable information in transcripts. Therapy logs data were clinical notes and therefore not appropriate for depositing in an archive.

References

1. Moore G, Audrey S, Barker M, et al. Process Evaluation of Complex Interventions: Medical Research Council Guidance. London: MRC Population Health Science Research Network; 2014.
2. Eccles MP, Mittman BS. Welcome to Implementation Science. Implementation Science. 2006;1(1):1.
3. Rogers PJ. Using programme theory to evaluate complicated and complex aspects of interventions. Evaluation. 2008;14(1):29-48.

For peer review only - http://bmjopen.bmj.com/site/about/guidelines.xhtml

4. Clare L, Kudlicka A, Oyebode JR, et al. Goal-oriented cognitive rehabilitation for early-stage Alzheimer's and related dementias: the GREAT RCT. Health Technol Assess. 2019;23(10).
5. Clare L, Kudlicka A, Bayer A, et al. Goal-oriented cognitive rehabilitation in early-stage Alzheimer's and related dementias: results from a multi-centre, single-blind, randomised controlled trial (The GREAT trial). Alzheimer's & Dementia: The Journal of the Alzheimer's Association. 2017;13(7):P899-P900.
6. Walton M. Applying complexity theory: a review to inform evaluation design. Eval Program Plann. 2014;45:119-26.
7. Greenhalgh T, Papoutsi C. Studying complexity in health services research: desperately seeking an overdue paradigm shift. BMC Med. 2018;16(1):95.
8. Callaghan G. Evaluation and negotiated order: developing the application of complexity theory. Evaluation. 2008;14(4):399-411.
9. Kernick D. Wanted—new methodologies for health service research. Is complexity theory the answer? Fam Pract. 2006;23(3):385-90.
10. Thompson DS, Fazio X, Kustra E, et al. Scoping review of complexity theory in health services research. BMC Health Services Research. 2016;16(1):87.
11. Paley J, Eva G. Complexity theory as an approach to explanation in healthcare: A critical discussion. International Journal of Nursing Studies. 2011;48(2):269-79.
12. Churruca K, Pomare C, Ellis LA, et al. The influence of complexity: a bibliometric analysis of complexity science in healthcare. BMJ Open. 2019;9(3):e027308.
13. Clark AM. What are the components of complex interventions in healthcare? Theorizing approaches to parts, powers and the whole intervention. Social Science & Medicine. 2013;93:185-93.
14. Braithwaite J, Churruca K, Long JC, et al. When complexity science meets implementation science: a theoretical and empirical analysis of systems change. BMC Med. 2018;16(1):63.
15. Caffrey L, Wolfe C, McKevitt C. Embedding research in health systems: lessons from complexity theory. Health Res Policy Syst. 2016;14(1):54.
16. Colón-Emeric CS, Lekan-Rutledge D, Utley-Smith Q, et al. Connection, regulation, and care plan innovation: a case study of four nursing homes. Health Care Manage Rev. 2006;31(4):337-46.
17. Chandler J, Rycroft-Malone J, Hawkes C, et al. Application of simplified Complexity Theory concepts for healthcare social systems to explain the implementation of evidence into practice. J Adv Nurs. 2016;72(2):461-80.
18. Trimmer JD. Response of Physical Systems. New York: Wiley & Sons; 1950.
19. Hawe P. Lessons from complex interventions to improve health. Annual Review of Public Health. 2015;36(1):307-23.
20. May CR, Johnson M, Finch T. Implementation, context and complexity. Implementation Science. 2016;11(1):141.
21. Hawe P, Shiell A, Riley T. Complex interventions: How "out of control" can a randomised controlled trial be? British Medical Journal. 2004;328:1561-3.
22. Kirk MA, Haines ER, Rokoske FS, et al. A case study of a theory-based method for identifying and reporting core functions and forms of evidence-based interventions. Transl Behav Med. 2019;11(1):21-33.
23. Byrne D. What Is complexity science? Thinking as a realist about measurement and cities and arguing for natural history. Emergence. 2001;3(1):61-76.
24. Eppel EA, Rhodes ML. Complexity theory and public management: a 'becoming' field. Public Management Review. 2018;20(7):949-59.
25. Clare L, Bayer A, Burns A, et al. Goal-oriented cognitive rehabilitation in early-stage dementia: study protocol for a multi-centre single-blind randomised controlled trial (GREAT). Trials. 2013;14(1):152.
26. Kudlicka A, Clare L. Cognitive rehabilitation in mild and moderate dementia. 2018. In: Oxford Research Encylopedia of Psychology [Internet].

For peer review only - http://bmjopen.bmj.com/site/about/guidelines.xhtml

27. Clare L, Kudlicka A. Goal-Oriented Cognitive Rehabilitation in Early-Stage Alzheimer's and related Dementias: Multi-Centre Single-Blind Randomised Controlled Trial (GREAT): Handbook for Therapists. Bangor: University of Bangor; 2015.
28. Craig P, Dieppe P, Macintyre S, et al. Developing and Evaluating Complex Interventions: New Guidance. London: MRC; 2008.
29. Warmoth K, Morgan-Trimmer S, Kudlicka A, et al. Reflections on a personalized cognitive rehabilitation intervention: Experiences of people living with dementia and their carers participating in the GREAT trial. Neuropsychological Rehabilitation. 2020:1-19.
30. Braun V, Clarke V. Thematic analysis. In: Cooper H, editor. APA Handbook of Research Methods in Psychology 2012. p. 57-71.
31. Ritchie J, Spencer L, O'Connor W. Carrying out qualitative analysis. In: Ritchie J, Lewis J, editors. Qualitative research practice: a guide for social science students and researchers. London, UK: SAGE Publications; 2003. p. 219-62.
32. Braun V, Clarke V. Using thematic analysis in psychology. Qual Res Psychol. 2006;3(2):77-101.
33. Maxwell JA. A Realist Approach for Qualitative Research. London: Sage; 2012.
34. Mitchell G, Agnelli J. Person-centred care for people with dementia: Kitwood reconsidered. Nursing Standard. 2015;30(7):46-50.
35. Delgado C, Upton D, Ranse K, et al. Nurses' resilience and the emotional labour of nursing work: An integrative review of empirical literature. International Journal of Nursing Studies. 2017;70:71-88.
36. Miller CJ, Wiltsey-Stirman S, Baumann AA. Iterative Decision-making for Evaluation of Adaptations (IDEA): A decision tree for balancing adaptation, fidelity, and intervention impact. J Community Psychol. 2020;48(4):1163-77.
37. Movsisyan A, Arnold L, Copeland L, et al. Adapting evidence-informed population health interventions for new contexts: a scoping review of current practice. Health Res Policy Syst. 2021;19(1):13.
38. Kirk AM. Adaptation. In: Nilsen P, Birken SA, editors. Handbook on Implementation Science. Cheltenham: Edward Elgar; 2020. p. 317-32.
39. Khan S, Vandermorris A, Shepherd J, et al. Embracing uncertainty, managing complexity: applying complexity thinking principles to transformation efforts in healthcare systems. BMC Health Services Research. 2018;18(1):192.
40. Long KM, McDermott F, Meadows GN. Being pragmatic about healthcare complexity: our experiences applying complexity theory and pragmatism to health services research. BMC Med. 2018;16(1):94.
41. Toomey E, Hardeman W, Hankonen N, et al. Focusing on fidelity: narrative review and recommendations for improving intervention fidelity within trials of health behaviour change interventions. Health Psychology and Behavioral Medicine. 2020;8(1):132-51.
42. Solman B, Clouston T. Occupational therapy and the therapeutic use of self. British Journal of Occupational Therapy;79(8):514-6.
43. Rahkonen T, Eloniemi-Sulkava U, Paanila S, et al. Systematic Intervention for Supporting Community Care of Elderly People After a Delirium Episode. Int Psychogeriatr. 2001;13(1):37-49.
44. Glanz K, Bishop DB. The role of behavioral science theory in development and implementation of public health interventions. Annual Review of Public Health. 2010;31(1):399-418.
45. Ling T. Evaluating complex and unfolding interventions in real time. Evaluation. 2012;18(1):79-91.
46. Gear C, Eppel E, Koziol-Mclain J. Advancing complexity theory as a qualitative research methodology. International Journal of Qualitative Methods. 2018;17(1).
47. Westhorp G. Using complexity-consistent theory for evaluating complex systems. Evaluation. 2012;18(4):405-20.

For peer review only - http://bmjopen.bmj.com/site/about/guidelines.xhtml

48. Grant RL, Hood R. Complex systems, explanation and policy: implications of the crisis of replication for public health research. Critical Public Health. 2017;27(5):525-32.
49. Rusoja E, Haynie D, Sievers J, et al. Thinking about complexity in health: A systematic review of the key systems thinking and complexity ideas in health. J Eval Clin Pract. 2018;24(3):600-6.
50. Brainard J, Hunter PR. Do complexity-informed health interventions work? A scoping review. Implementation Science. 2016;11(1):127.

For peer review only

Appendix 1: Methods and findings reported in accordance with the Standards for Reporting Qualitative Research (SRQR)

S1 Title	The title indicates the type of study and theoretical focus of the paper
S2 Abstract	An abstract with background, methodological detail and conclusions has been included
S3 Problem formulation	The introductory section comments on limitations of process evaluations and outlines how complexity theory could improve process evaluations
S4 Purpose or research question	The research question is stated in the first paragraph of the introduction.
Methods	
S5 Qualitative approach and research paradigm	The methods section states that the paper presents findings from a process evaluation embedded in a trial. The methods section describes the qualitative research as underpinned by a critical realist paradigm, and that qualitative interview and focus group data were analysed using a combined deductive and inductive approach.
S6 Researcher characteristics and reflexivity	The methods section states that the researchers analysing the data had no previous involvement in the study
S7 Context Setting/site and salient contextual factors	The introduction and background section provides information about the intervention setting, and includes references to more detailed publications about the study setting.
S8 Sampling strategy How and why research participants, documents, or events were	The methods section describes how participants were sampled. All available therapists were invited to a focus group, people with dementia were consecutively sampled across different sites, and therapy logs were sampled for around 10% of people receiving the intervention who had the best and worst outcomes from the intervention.

For peer review only - http://bmjopen.bmj.com/site/about/guidelines.xhtml

S9 Ethical issues	Details and reference number of the ethics approval are included
S10 Data collection methods	The timing and methods for data collection are described in detail in the methods section
S11 Data collection instruments	Data collection instruments are included in the appendices
S12 Units of study	Numbers and descriptions of participants are included
S13 Data processing	Transcription and use of NVivo are noted in the methods section. All data were stored on secure university servers.
S14 Data analysis	The method and stages of data analysis are described. The analysis of therapy logs drew on findings from the focus group – this is included in the description of methods.
S15 Techniques to enhance trustworthiness	The methods section states that focus group and interview data were collected by researchers without close involvement in the intervention. Data were analysed by researchers who had not been involved in the study up to that point (SMT and KW); developing themes from all datasets were discussed in meetings by the research team
Results/findings	
S16 Synthesis and interpretation	Findings are presented in relation to the theoretical concepts described in the introductory section.
S17 Links to empirical data	Quotes are included to substantiate the themes presented in the findings section
Discussion	
S18 Integration with prior work, implications, transferability, and contribution(s) to the field	Findings are summarised in the discussion section. Findings are also related to future implementation of the intervention and implications for the theoretical perspective (complexity theory)

For peer review only - http://bmjopen.bmj.com/site/about/guidelines.xhtml

S19 Limitations Trustworthiness and limitations of findings	Limitations of the study are included in the discussion section.
S20 Conflicts of interest	The authors have declared there are no conflicts of interest.
S21 Funding	The funder and grant number is stated.

For peer review only

For peer review only - http://bmjopen.bmj.com/site/about/guidelines.xhtml

Appendix 2: GREAT focus group schedule - therapists' experience of providing goal-oriented cognitive rehabilitation for people with dementia (PwD) in GREAT

Introduction - aims of the focus group

The aim of this focus group is to explore therapists' experience of working with PwD to provide goal-oriented CR, to capture the nature of the interactions and the way in which the therapists deploy their skills to achieve the desired outcomes. We will use the results to inform the process of educating others on how to implement goal-oriented CR and to consider ways to further enhance CR in future.

- We value your open and honest responses. Please feel free to share your point of view even if it differs from what others have said. Every person's experiences and opinions are important.
- We are tape recording to capture everything you have to say, but we won't identify anyone by name in the report. The recording will be transcribed by someone from outside the team and anonymised.

General views on the process of providing goal-oriented cognitive rehabilitation – 10 min

How do you find the process of providing goal-oriented CR in GREAT?

What is it like to provide goal-oriented CR in GREAT?

Things that have worked well in providing goal-oriented CR – 10 min

What are the things that work well for you in the therapy process?

Which aspects do you look forward to most?

Challenges or difficulties in providing goal-oriented CR –10 min

What aspects of your work on providing goal-oriented CR in GREAT are most challenging?

What are the difficulties that you encounter when providing goal-oriented CR in GREAT?

Additional questions on the process of providing goal-oriented cognitive rehabilitation – 15 min

What sort of things have you learnt so far about working on cog rehab goals from working on the trial?

How do you find the role of the carer during the therapy process?

Has anything surprised you about the process?

Do you have hunches about what sort of people seem to benefit most?

If you were in charge, is there anything you'd be tempted to change about the way we have set up the study regarding working on the goals?

What do you think about the length of sessions?

What do you think about the overall length of the therapy?

How do you find training and supervision provided in GREAT?

If you were providing top tips to a therapist, what would be the key things you'd advise them to look out for or do?

Closing question

Before we finish the focus group, is there anything that we have missed? Is there anything anyone would like to say about their experience of providing goal-oriented CR in GREAT?

For peer review only - http://bmjopen.bmj.com/site/about/guidelines.xhtml

Summing up and moving on

Summarise main points of the discussion, balance negative comments with positive comments. Thank everyone for sharing their comments on providing goal-oriented CR in GREAT and end the focus group.

For peer review only

For peer review only - http://bmjopen.bmj.com/site/about/guidelines.xhtml

Appendix 3: Topics recorded by therapists in therapy logs

<u>**Therapy log topic**</u>	<u>**Therapist ratings or comments recorded under relevant sessions**</u>
Compliance	Whether given session was completed
Relationships	The relationship the therapist developed with the participant and carer, and the relationship between the participant and carer
Goals	Participant and carer responsiveness to the solution-focused problem-solving approach<br>Goal 1: agreed strategies, between-session practice, and progress<br>Goal 2: agreed strategies, between-session practice, and progress<br>Goal 3: agreed strategies, between-session practice, and progress<br>Any adjustments or modifications to goal statements provided at baseline<br>In-session goal attainment ratings by participant, carer and therapist (sessions 10 and 14) and comments on these<br>Selection of goal attainment scaling indicators for each goal (sessions 10 and 14) and comments on these
Activity levels	Review of activity levels, plan for behavioural activation to increase activity engagement, and comments on progress
Compensatory strategy use	Review of current use of compensatory strategies and environmental adaptations, plan to develop strategy use, and comments on progress
Restorative strategy use	Response to information about restorative strategies, plan for developing restorative strategy use, and comments on progress
Attention and concentration	Strategies introduced to help maintain attention and concentration, and progress with applying these strategies

For peer review only

For peer review only - http://bmjopen.bmj.com/site/about/guidelines.xhtml

Anxiety management	Current use of anxiety management strategies, carer's perspective on the participant's use of these strategies, introduction or refinement of anxiety management technique(s), and progress with use of anxiety management strategies
Carer well-being	Review with carer, and plan for enhancing carer well-being
Carer involvement	The extent to which the carer was engaged in supporting the process of therapy
Ending therapy	Plans for maintaining progress after the end of the intervention, and review of other sources of help and support
Experience of therapy	Review with the participant and with the carer, and therapist reflection on the process of therapy; therapist confidence in addressing participants' goals (following the nine month follow-up)

For peer review only

For peer review only - http://bmjopen.bmj.com/site/about/guidelines.xhtml

Appendix 4: Interview schedule for the person with dementia

The interview will take form of a conversation and the interviewer will encourage the participant to talk freely about the experience of the cognitive rehabilitation intervention.

The researcher will begin by re-establishing consent for the interview and for audio-recording.

The researcher may begin with some general conversation to build rapport as appropriate.

The researcher will introduce the main part of the interview by saying:
You've been taking part in the GREAT study and having visits from the therapist, and I'd like to know your views on what it was like. I'm interested in what it was like to take part in the study and how you found the visits from the therapist.

1. Experiences
The researcher will explore the participant's experiences and feelings starting with general questions:
How did you find the therapist's visits over the past few weeks?
What was it like to work with the therapist on your goals?

More specific information will then be elicited using prompts such as the following:
What were the more enjoyable things about your work with the therapist?
What were the less enjoyable things about your work with the therapist?
What aspects of your work with the therapist were more/less helpful?
Did you find it hard work to take part in the therapy?
What aspects of your work with the therapist were most challenging?
The interviewer will encourage the person to give specific examples, where possible.

2. Outcomes
The researcher will explore the impact of taking part in cognitive rehabilitation on the person's everyday life and self-perceptions.

What difference (if any) has your work with the therapist made to your daily life?
Has the experience changed anything in the way you think about your dementia/about yourself/about the future?
Has the experience changed anything in the way you relate to your carer/family?

The researcher will draw on positive comments from the participant to end the conversation on a positive note.

For peer review only - http://bmjopen.bmj.com/site/about/guidelines.xhtml
